# Advancements in Radiomics-Based AI for Pancreatic Ductal Adenocarcinoma

**DOI:** 10.3390/bioengineering12080849

**Published:** 2025-08-06

**Authors:** Georgios Lekkas, Eleni Vrochidou, George A. Papakostas

**Affiliations:** MLV Research Group, Department of Informatics, Democritus University of Thrace, 65404 Kavala, Greece; gelekka@cs.duth.gr (G.L.); evrochid@cs.duth.gr (E.V.)

**Keywords:** artificial intelligence, radiomics, pancreatic ductal adenocarcinoma, medical imaging, radiogenomics, medical image analysis, computer vision

## Abstract

The advancement of artificial intelligence (AI), deep learning, and radiomics has introduced novel methodologies for the detection, classification, prognosis, and treatment evaluation of pancreatic ductal adenocarcinoma (PDAC). As the integration of AI into medical imaging continues to evolve, its potential to enhance early detection, refine diagnostic precision, and optimize treatment strategies becomes increasingly evident. However, despite significant progress, various challenges remain, particularly in terms of clinical applicability, generalizability, interpretability, and integration into routine practice. Understanding the current state of research is crucial for identifying gaps in the literature and exploring opportunities for future advancements. This literature review aims to provide a comprehensive overview of the existing studies on AI applications in PDAC, with a focus on disease detection, classification, survival prediction, treatment response assessment, and radiogenomics. By analyzing the methodologies, findings, and limitations of these studies, we aim to highlight the strengths of AI-driven approaches while addressing critical gaps that hinder their clinical translation. Furthermore, this review aims to discuss future directions in the field, emphasizing the need for multi-institutional collaborations, explainable AI models, and the integration of multi-modal data to advance the role of AI in personalized medicine for PDAC.

## 1. Introduction

Pancreatic cancer (PC) is the third-leading cause of cancer death in men and women combined. As pancreatic malignant tumors are aggressive, the 5-year survival rate is hovering at around 5–10%. Such dire statistics are largely attributed to poor prognosis and limited treatment options. Early detection is critical for improving patient outcomes, as the only curative therapeutic option is surgical resection [1]. Given the aggressive nature of pancreatic cancer, many tumors are diagnosed at an advanced stage where surgical resection is not possible or tumor metastasis has taken place. Consequently, there is a pressing need for innovative methods to enhance diagnostic accuracy and increase early detection.

Radiomics has emerged as a promising field that capitalizes on advanced image analysis to quantify tumor phenotypes. Originally developed as a way to mine high-dimensional data from standard medical images, radiomics allows researchers and clinicians to extract and interpret complex textural, shape-based, and intensity-based features. In pancreatic cancer, radiomic techniques have shown potential in discriminating between malignant and benign lesions, predicting treatment response, and stratifying patient prognosis [2]. By transforming medical images into a dataset of quantifiable metrics, radiomics hold the promise of uncovering imaging biomarkers that can guide personalized clinical decision-making.

Parallel to the rise in radiomics, the field of artificial intelligence (AI) has experienced rapid growth, particularly with the advent of deep learning approaches. Deep learning, rooted in neural networks (NNs) capable of automatically learning feature representations, has revolutionized image-based tasks in medical applications [3]. From detecting microcalcifications in mammograms to segmenting brain tumors in MRI scans, AI-driven models—especially convolutional neural networks (CNNs)—have demonstrated a human-level performance in various diagnostic contexts. When applied to pancreatic cancer, these systems aim to identify early signs of neoplasia and differentiate subtle morphological patterns that may be challenging for human observers to detect [4].

An emerging and increasingly explored concept is the combination of radiomics with deep learning, sometimes referred to as “deep radiomics” [5]. The rationale behind this integration is clear: while radiomics provide handcrafted, interpretable features that reflect known statistical or geometric properties, deep learning can uncover latent patterns and relationships directly from data without explicit feature engineering. By merging these complementary approaches, researchers hope to maximize predictive performance, improve model robustness, and expand the range of discovered imaging biomarkers. This synergistic strategy could enhance early pancreatic cancer detection, refine prognostic assessments, and facilitate the development of adaptive treatment protocols [6,7].

To this end, this work presents a comprehensive review of advancements in artificial intelligence and radiomics for pancreatic ductal adenocarcinoma (PDAC). We place particular emphasis on studies that explore the integration of radiomic features with machine learning and deep learning methods to improve diagnostic and prognostic accuracy. Rather than offering a broad overview, this review aims to provide a more focused and in-depth analysis of how handcrafted radiomic features and learned representations have been applied, individually and in combination, across a range of clinical applications. We also highlight the role of multi-modal imaging, multi-omics approaches such as radiogenomics, and advanced preprocessing pipelines to offer a detailed understanding of current methodologies, challenges, and opportunities in the field.

The rest of this work is structured as follows: Section 2 reviews related works and highlights the specific contributions of this study. Section 3 presents the research methodology. Section 4 provides a detailed overview of the literature organized around clinical applications, including disease classification, detection, survival prediction, treatment response, and radiogenomics, along with a focused discussion on deep radiomics approaches due to their broad applicability across multiple clinical tasks. Section 5 summarizes the datasets, commonly used features, and methodologies reported in the literature. Section 6 discusses the role of AI, deep learning, and radiomics in PDAC, along with current challenges and limitations. Finally, Section 7 concludes the paper.

## 2. Related Works and Contributions

Several recent reviews have explored the application of radiomics and artificial intelligence (AI) methods in pancreatic cancer imaging, each offering distinct insights but also exhibiting limitations that our current review addresses comprehensively.

Casà et al. (2022) [8] examined the impact of radiomics on diagnosing and staging pancreatic malignancies, with a strong emphasis on feature selection and validation. However, their focus was confined to CT-based staging and offered little coverage of deep learning or cross-modality fusion methods, leaving significant gaps that our review addresses by integrating multi-modality (CT, MRI, and PET) approaches. Castiglioni et al. (2021) [9] discussed the evolution of AI in medical imaging from conventional machine learning to deep learning, outlining the technical advances but not specifically focusing on pancreatic imaging or the synergy between handcrafted radiomics features and CNN-based embeddings. Building upon their technical overview, our review instead tailors these advanced architectures to pancreatic cancer, showing how radiomics can be merged with learned representations for improved diagnostic accuracy.

Guiot et al. (2021) [10] offered a generalized discussion on radiomics for personalized medicine across various disease sites, including broad workflow descriptions and potential applications. Yet they did not delve into the unique aspects of pancreatic cancer detection and classification, nor did they explore deep learning–radiomics fusion in depth—both central themes of our work. Yao et al. (2023) [11] provided a more pancreas-specific survey, reviewing convolutional neural networks and basic radiomics methods but lacking a thorough exposition of how handcrafted radiomic and deep learning features can be effectively combined. Our review aims to fill that gap by demonstrating explicit fusion strategies, detailing interpretability mechanisms, and illustrating robust external validation steps.

Huang et al. (2024) [12] reviewed AI applications primarily for tumor detection, segmentation, and prognosis in pancreatic cancer, underscoring high sensitivities that approached or matched expert radiologists. Despite this valuable insight into AI’s potential, they did not substantially address the notion of fusing radiomics with deep learning embeddings. By contrast, we systematically review such integrative models, highlighting the ways in which combining handcrafted and learned features can boost performance across multiple imaging modalities. Hayashi et al. (2021) [13] framed artificial intelligence in the context of pancreatic ductal adenocarcinoma (PDAC), focusing on microenvironmental factors and early detection via omics-based approaches. Nevertheless, they excluded benign pancreatic conditions and largely omitted multi-modal or fusion pipelines. Here, we expand to include benign and cystic lesions and examine advanced model pipelines that unify radiomics with deep learning to meet real-world challenges.

Abunahel et al. (2020) [14] conducted a systematic review specifically targeting pancreas-based radiomics, identifying reproducibility issues and commonly used imaging features but stopping short of addressing deep learning or fusion frameworks. Our work bridges that shortfall by incorporating these cutting-edge methods, exploring how radiomics can be augmented with deep neural networks to tackle detection, classification, and survival endpoints. A more introductory paper by Ahmed et al. (2023) [15] presented fundamental AI concepts (e.g., convolutional architectures, training paradigms), serving as a helpful primer but offering limited discussion on large-scale external validations, multi-omics, or the synergy between handcrafted radiomics and CNNs. Our review, therefore, delves into these advanced topics, demonstrating how multi-institutional data harmonization and integrative pipelines can address heterogeneity and interpretability concerns. Finally, Avanzo et al. (2017) [16] laid out key concepts in radiomics, highlighting early insights into reproducibility and the potential for large-scale feature extraction in oncology. Although foundational, their work preceded modern deep learning-based feature extraction and synergy with radiomics specifically for pancreatic cancer, which our current review covers extensively.

To this end, in this review, we included studies that used radiomics or artificial intelligence techniques to study pancreatic cancer, while also applying more stringent criteria to identify and analyze studies that implemented fusion models, which are approaches that combine radiomics with machine learning or deep learning to improve diagnostic and prognostic accuracy. While previous reviews have provided broad overviews of AI-based methods or radiomics in isolation, we place a greater emphasis on exploring fusion models in depth, investigating how the combination of handcrafted radiomic features and learned representations can enhance accuracy, interpretability, and robustness. We also considered multi-modal imaging data (CT, MRI, and PET), multi-omics (radiogenomics), and advanced preprocessing pipelines to provide a comprehensive view of cutting-edge research. This approach allowed us to highlight developments in emerging applications such as subregional texture analysis, automated tumor segmentation, and multi-institutional external validations.

## 3. Research Methodology

A comprehensive literature search was performed using the PubMed database by combining the subject terms “Radiomics”, “Machine Learning”, “Deep Learning”, “Artificial Intelligence”, and their associations with “Pancreatic Cancer”, “PDAC”, “Tumor Detection”, “Classification”, “Survival Prediction”, “Treatment Response”, “Radiogenomics”, and “Fusion Models”. The initial search took place in March 2025, and the aim was to identify original research articles focusing on radiomics and AI-related methods applied to pancreatic cancer.

We applied the following inclusion criteria:

1. Studies that implemented radiomics or deep learning-based radiomic analyses.
2. Investigations covering uni- or multi-modality imaging approaches (e.g., CT, MRI, and PET).
3. Fusion models integrating radiomics with machine learning or deep learning techniques.
4. Research exploring single- or multi-omics (e.g., radiogenomics).
5. English-language publications that reported human subjects’ data on pancreatic cancer.

All titles and abstracts were screened initially to exclude off-topic work. Articles deemed relevant then underwent an independent full-text review to confirm eligibility based on the research scope. Exclusion criteria encompassed studies from unrelated fields, non-English publications, review articles, conference abstracts, case reports, duplicate citations, and articles lacking human data in the context of pancreatic cancer radiomics.

It should be noted that PubMed was chosen exclusively due to its reliable coverage of peer-reviewed biomedical studies, ensuring that the included studies were clinically relevant, human-subject focused, and of high scientific quality. Given our emphasis on translational applications of AI in medical imaging and oncology, PubMed offered a rigorous and curated source most aligned with our review’s scope. While multi-database research may have captured additional publications, we prioritized clinical depth and reproducibility over broader coverage, believing this strategy could sufficiently support the objectives of our review.

**Figure 1:**
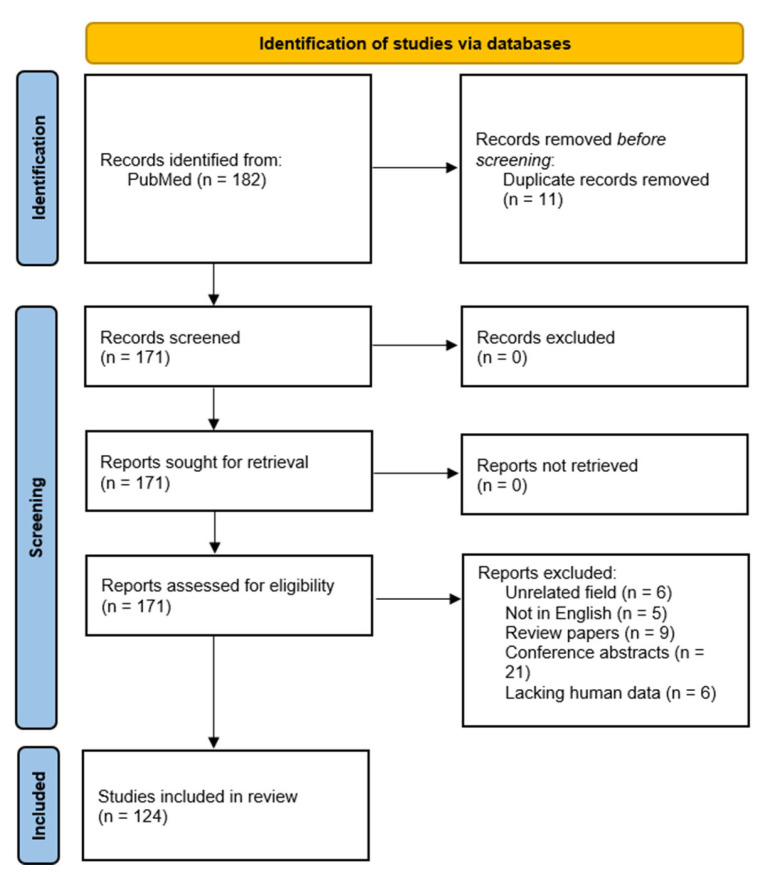
The PRISMA 2020 flow diagram.

**Figure 2:**
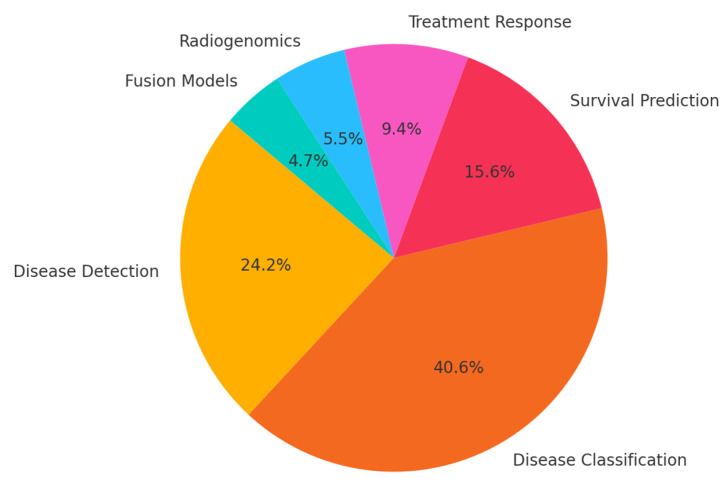
Topics of the selected literature.

Our search resulted in 124 papers. The PRISMA 2020 standard was followed, including the extensive exclusion criteria mentioned above. The PRISMA 2020 flow diagram is illustrated in Figure 1. A pie chart of the papers regarding their topic of focus is illustrated in Figure 2. As it can be observed, the main topics identified in the literature are as follows: disease classification, disease detection, survival prediction, treatment response, radiogenomics, and deep radiomics fusion models. In what follows, a taxonomy is applied based on these topics (clinical applications) towards providing a comprehensive review of related studies. It should be clarified that deep radiomics is a methodological approach, yet in this work, it is placed within clinical applications due to the fact that deep radiomics is increasingly applied in real-world clinical tasks.

## 4. Comprehensive Review of the Literature by Clinical Application

### 4.1. Disease Classification

He et al. (2019) [17] differentiated atypical non-functional pancreatic neuroendocrine tumors (NF-pNETs) from PDAC in 147 patients using a CT-based radiomics framework with 647 extracted features, ultimately retaining 7 through LASSO selection. Their integrated model (clinicoradiological + radiomics) achieved an AUC of 0.884, outperforming a clinicoradiological approach alone (0.775). However, the study itself suggested some limitations that may have affected their results. According to the author, the study was retrospective and only images in 5 mm thickness were available. Turning to cystic lesions, Xie et al. (2020) [18] analyzed 57 cases (31 mucinous cystic neoplasms, 26 macrocystic serous cystadenomas) and extracted 1942 CT texture features. By combining conventional radiological and radiomics information, they reached 98.2% accuracy (AUC = 0.994). On the drawback, the author pointed out some limitations that may have affected the results of their study. A selection bias may have been introduced by the inclusion criteria, the whole study was performed on one single manufacturer, and only portal-phase images were analyzed. Mashayekhi et al. (2020) [19] then distinguished functional abdominal pain, recurrent acute pancreatitis, and chronic pancreatitis in 56 patients using 54 radiomic features, ultimately selecting 11 key predictors and achieving an 82.1% overall accuracy with a one-vs-one IsoSVM. Meanwhile, Attiyeh et al. (2018) [20] focused on 103 preoperative CT scans of branch duct IPMNs, extracting 256 features and employing a Random Forest that improved risk stratification (AUC = 0.79) when quantitative mural nodularity features were added.

Liu et al. (2022) [21] integrated multiparametric MRI radiomics (6 selected features from 960) with clinical biomarkers (CA19-9, CEA) in 102 patients (54 PC, 48 MFCP), boosting the AUC to 0.960 versus 0.799 for clinical data alone. Kulali et al. (2018) [22] used DWI-MRI in 30 nonfunctional PanNET patients, showing high-grade tumors had significantly lower ADC values, aiding preoperative tumor grading. Park et al. (2020) [23] applied a radiomics-based Random Forest to 182 dual-phase CTs (89 autoimmune pancreatitis, 93 PDAC), attaining a 95.2% accuracy (AUC = 0.975) in distinguishing AIP from PDAC. Lastly, Wei et al. (2019) [24] used an SVM with 22 selected features (from 409) to differentiate serous cystic neoplasms from other pancreatic cystic neoplasms in 260 MDCT scans, achieving an AUC of 0.767 (cross-validation) and 0.837 (independent validation)—all reinforcing the value of radiomics-driven machine learning for more precise, non-invasive pancreatic lesion characterization.

Reinert et al. (2020) [25] investigated 95 patients (53 PDAC, 42 PNEN) using portal-venous phase CT (Siemens SOMATOM), extracting 92 radiomic features with PyRadiomics. They identified eight key first-/second-order texture features (e.g., median, energy, and GLCM) and built a multivariate logistic regression model that achieved a 75.8% accuracy (sensitivity 79.2%, specificity 71.4%). However, the author pointed out some limitations that may have affected the results in their study. Image data were collected on different multi-slice scanners, but they used a similar examination and contrast agent injection protocol. Moreover, morphologic imaging features were not evaluated in the study. Next, Polk et al. (2020) [26] examined 51 IPMN cases (29 malignant, 22 benign) with multiphase CT, extracting 39 radiomic features per phase. Their final radiomics + International Consensus Guidelines (ICG) model reached an AUC of 0.93 (5-fold cross-validation AUC = 0.90), outperforming ICG alone (AUC = 0.817). Focusing on MRI, Flammia et al. (2023) [27] studied 50 BD-IPMN patients (31 low-risk, 19 high-risk) with at least two contrast-enhanced scans, extracting 107 features per sequence. Using LASSO, they obtained up to AUC = 0.99 (T1W pre-contrast) for predicting malignant potential. Meanwhile, Benedetti et al. (2021) [28] analyzed 39 PanNET patients’ pre-surgical CT scans, extracting 69 radiomic features (ceCT + non-ceCT) via MATLAB. Sphericity (AUC = 0.79), tumor volume, and voxel-alignment features were key predictors for grading and metastasis risk. Tikhonova et al. (2023) [29] then assessed 91 PDAC patients on arterial/portal/delayed phases using LifEx (376 texture features), selecting 5 via LASSO and achieving an AUC = 0.75 for grade ≥ 2 tumors.

Zhang et al. (2022) [30] targeted 138 chronic pancreatitis patients (67 MFCP, 71 PDAC) who underwent contrast-enhanced multidetector CT; after manual 3D Slicer segmentation and LASSO-driven feature selection, their radiomics-based nomogram reached an AUC up to 0.93. Kim et al. (2015) [31], in contrast, used no radiomics but examined 167 lesions from 161 pancreatic neuroendocrine neoplasm patients with dynamic ceCT. They found a portal enhancement ratio < 1.1 plus imaging features (e.g., poorly defined margins, bile duct dilatation) accurately distinguished Grade 3 NEC (92.3% sensitivity, 80.5% specificity). However, the author reported some limitations. In their study, various CT scanners were used with a relatively thick reconstruction thickness. Additionally, two contrast agents with different concentrations were used. Li et al. (2023) [32] automated tumor segmentation in 512 MRI scans (123 PASC, 389 PDAC) to extract radiomic features with PyRadiomics; their mixed linear discriminant model achieved an AUC = 0.94 in validation, outperforming clinical-alone or radiomics-alone approaches. Lastly, Chu et al. (2022) [33] investigated 214 patients with confirmed PCNs (IPMNs, MCNs, SCAs, SPNs, and PanNETs), extracting 488 venous-phase ceCT features. A Random Forest classifier attained an AUC = 0.940, surpassing an expert radiologist’s AUC = 0.895, thereby underscoring the capacity of radiomics-based AI to enhance pancreatic lesion diagnosis.

Yang et al. (2022) [34] retrospectively analyzed 110 patients (63 SCNs, 47 MCNs, including 3 mucinous cystadenocarcinomas) who underwent unenhanced and enhanced CT, using both manual and semi-automatic rectangular segmentation to generate single- and multi-channel inputs. By comparing multiple feature extraction methods (wavelet, LBP, HOG, GLCM, Gabor, ResNet, and AlexNet) and classifiers (KNN, Softmax, Bayes, and Random Forest), their Multi-channel-Multiclassifier-Random Forest-ResNet (MMRF-ResNet) model achieved the best performance, with AUC = 0.98, 91.63% sensitivity, 93.80% specificity, and 92.69% accuracy. In a similar context, Chen et al. (2021) [35] employed a CT-based radiomics nomogram in 89 patients (31 SCNs, 30 IPMNs, and 28 MCNs), extracting 710 features from plain, arterial, and portal phases. LASSO selection plus logistic regression yielded a three-phase radiomics signature (AUC = 0.960 in training, 0.817 in validation), further enhanced when integrated with clinical features. Bian et al. (2021) [36] investigated MRI-based radiomics in 157 surgically confirmed non-functioning pancreatic neuroendocrine tumors (NF-pNETs), extracting arterial and portal phase features and using LASSO to build a seven-feature rad-score linked to tumor grading (AUC = 0.775). Focusing on unenhanced CT, Ren et al. (2020) [37] examined 109 patients (30 MFP, 79 PDAC) and balanced the dataset via SMOTE (90 MFP, 120 PDAC). Of the 396 extracted features, four selected by mRMR powered a Random Forest model with 93.3% accuracy (LGOCV sensitivity = 82.6%, specificity = 80.8%). The limitations referred to are that, due to the nature of their study, a retrospective study, selection bias was implemented. The used dataset was relatively small, while two different scanners were used. Van der Pol et al. (2019) [38] considered 43 resected PNETs vs. 28 resected pancreatic RCC metastases across multiple centers, noting that PNETs were larger, more often calcified, and had higher texture entropy (6.32 vs. 5.96, *p* = 0.004). Their final model (tumor size + entropy) reached an AUC of 0.77.

Zhang et al. (2023) [39] tackled 143 pathologically confirmed PCNs (SCNs, MCNs, and IPMNs), splitting them into development (*n* = 102) and test (*n* = 41) cohorts, extracting 1218 radiomics features, and achieving up to an 80.4% accuracy for multiclass classification via a Random Forest. Meanwhile, Chang et al. (2020) [40] analyzed 301 PDAC patients (split 151/150 for training/test, plus 100 external) using IBEX to extract parenchymal-phase CT features and build a LASSO-based radiomics signature, reporting AUCs of 0.961/0.910 internally and 0.770 externally for histological-grade prediction. Turning to endoscopic ultrasound, Zhu et al. (2013) [41] used EUS images from 388 patients (262 PC, 126 CP), extracting 105 texture features, selecting 16 with SFS, and training an SVM that averaged a ~94.26% accuracy. Săftoiu et al. (2012) [42] examined EUS elastography in 258 patients (211 pancreatic adenocarcinoma, 47 chronic pancreatitis) across 13 European centers, using a multilayer perceptron ANN on hue histogram data and achieving a 91.14% training accuracy (84.27% testing) with AUC = 0.94. Lastly, Kang et al. (2015) [43] differentiated pancreatic RCC metastases (37 lesions) from hypervascular PNETs (31 lesions) in 44 patients using arterial and portal-phase CT; assessing relative percentage washout (RPW), they found a cutoff of 19% that yielded a ~84% accuracy, demonstrating a non-invasive means of discriminating pRCC from pNETs.

Hanania et al. (2016) [44] retrospectively analyzed 53 surgically confirmed IPMNs (34 high-grade, 19 low-grade), extracting 360 CT-based intensity, texture, and shape features. GLCM-based texture metrics proved most discriminative, and a 14-feature logistic regression model achieved AUC = 0.96, outperforming the Fukuoka criteria. Proietto Salanitri et al. (2022) [45] then applied a Vision Transformer (ViT) to MRI scans from 139 patients (normal, low-grade dysplasia, high-grade dysplasia, and adenocarcinoma), surpassing CNNs (AlexNet, DenseNet-121, ResNet18, EfficientNet-b5, and MobileNet-v2) with 70% accuracy. Focusing on malignancy classification, Gai et al. (2022) [46] studied 77 CECT scans (33 malignant, 44 benign), extracting 1267 features via MaZda, selecting 12 via LASSO, and employing an SVM with leave-one-case-out cross-validation to reach AUC = 0.750. Meanwhile, Pawlik et al. (2008) [47] analyzed data from 203 patients at a single-day multidisciplinary clinic, finding that 23.6% of treatment plans changed, highlighting the clinical impact of expert re-review.

**Figure 3:**
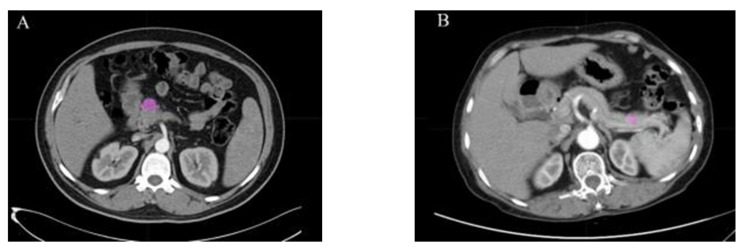
Examples of (**A**) a 51-year-old male with PDAC with a lesion in the pancreatic head; (**B**) a 60-year-old female with pNET with a lesion in the pancreatic body. Pink areas in the image correspond to the delineated ROIs [49].

Chakraborty et al. (2018) [48] investigated 103 BD-IPMNs on preoperative CECT, extracting both standard texture and radiographically inspired features (EBF, EIF, etc.). A Random Forest combining imaging and clinical data reached AUC = 0.81, improving on radiomics alone (AUC = 0.77). Shifting to tumor subtypes, Zhang et al. (2022) [49] differentiated PDAC (*n* = 156) from pNET (*n* = 82) using 48 LIFEx features. Testing 45 feature selection/classifier pairs, they found the GBDT+Random Forest optimal (AUC = 0.930 in validation). Figure 3 presents an example of disease classification, illustrating the differentiation of PDAC and pNET lesions. Ma et al. (2022) [50] then analyzed 175 patients (151 PC, 24 CP) with 1037 CECT radiomics features and clinical biomarkers; their combined (arterial + venous) logistic model achieved AUC = 0.980. Finally, Vaiyapuri et al. (2022) [51] proposed IDLDMS-PTC for 500 CT images (250 with pancreatic tumors, 250 controls), merging Emperor Penguin Optimizer-based segmentation and a MobileNet autoencoder. Their approach attained 99.35% accuracy, surpassing traditional CNN variants, and underscoring how deep learning-driven strategies can dramatically bolster pancreatic pathology classification.

Wang et al. (2022) [52] examined 139 PNET patients (83 training, 56 validation) using up to 1133 radiomics features from triple-phase CT scans, selecting eight via LASSO and integrating them with T-stage and MPD/BD dilation in an SVM-linear model; their best nomogram (plain-phase CT) achieved an AUC of 0.919 (training) and 0.875 (validation). The author pointed out, as a major limitation, the relatively insufficient sample size of just 139 PNET patients. Shifting to MRI, Shi et al. (2020) [53] assessed 66 cases (31 PNETs, 35 SPTs) with T2WI and DKI (Dapp, Kapp) sequences, extracting 195 features and using a logistic model (incorporating age, sex, and radiomics signature) that reached an AUC of 0.97 (training) and 0.86 (validation).

Focusing on mass-forming pancreatitis vs. PDAC, Ren et al. (2019) [54] evaluated 109 CECT scans (30 MFP, 79 PDAC), extracting 396 features; a combined model (SurfaceArea, Percentile40, LongRunEmphasis, and GLCMEntropy) delivered a 0.98 AUC, with 94% sensitivity and 92% specificity. For cystic lesions, Yang et al. (2019) [55] studied 78 patients (53 SCAs, 25 MCAs) via LIFEx-derived texture features, training a Random Forest that achieved an AUC of 0.77 (training) and 0.66 (validation) with 2 mm slices and 0.75 for 5 mm in validation.

Turning to deep learning, Li et al. (2019) [56] employed DenseNet on 206 CECT scans spanning four subtypes (IPMN, MCN, SCN, and SPT), omitting manual segmentation altogether; the model attained a 72.8% overall accuracy. Bevilacqua et al. (2021) [57] used [68Ga]Ga-DOTANOC PET/CT in 51 PanNET patients, extracting first-/second-order SUV features. Their best model, focusing on normalized homogeneity and entropy, reached an AUC of 0.90. Similarly, Gu et al. (2019) [58] recruited 138 PNETs from two institutions, extracting 853 arterial/venous-phase features, then fusing them into a radiomics signature integrated with a tumor margin. This nomogram yielded an AUC of 0.974 (training) and 0.902 (validation).

Kuwahara et al. (2019) [59], meanwhile, deployed a ResNet50-based CNN on 3970 EUS images from 50 IPMN patients (27 benign, 23 malignant), achieving AUC = 0.98, notably surpassing human diagnosis (56% accuracy). Limitations included the nature of the study, which was retrospective and single-center. Moreover, the sample size was relatively small, while the authors tried to overcome this issue with a 10-fold cross-validation technique. Finally, Tobaly et al. (2020) [60] analyzed 408 IPMN patients (181 low-grade, 128 high-grade, and 99 invasive), extracting 107 features via PyRadiomics and training a LASSO logistic regression. Their radiomics-only approach reached AUC = 0.84 (training) and 0.71 (validation), while including surgical indication variables boosted the validation AUC to 0.75—further underscoring the utility of radiomics for IPMN risk stratification.

Li et al. (2018) [61] retrospectively compared 127 patients with PDAC (*n* = 50) vs. pNETs (*n* = 77), extracting histogram-based texture features (e.g., percentiles, skewness) from portal-phase CT using FireVoxel. Their top combination (fifth percentile + skewness) reached an AUC = 0.887 (90% sensitivity, 80% specificity) for distinguishing atypical pNETs. In another risk stratification approach, Hernandez-Barco et al. (2023) [62] analyzed 575 resected IPMN patients (53.4% low-grade) using 18 clinical/imaging variables in a linear SVM (IPMN-LEARN), scoring an AUC = 0.82 with 77.4% accuracy, potentially reducing unnecessary IPMN surgeries.

Moving to MRI, Cui et al. (2021) [63] studied 202 BD-IPMN patients across three centers, extracting 1312 T1-w/T2-w/CET1-w features and selecting 9 via LASSO. Their radiomic signature, integrated with CA19-9 and MPD size, yielded AUCs up to 0.903 (training) and 0.884/0.876 (two external validations). Guo et al. (2018) [64] compared 42 CECT scans (28 PDAC, 14 PNEC) and found PNECs had better defined margins, less parenchymal atrophy, and distinct texture (lower entropy, higher uniformity), with arterial/portal contrast ratios showing AUC = 0.98–0.99 for discrimination.

Tong et al. (2022) [65] pivoted to a contrast-enhanced ultrasound (CEUS), applying a ResNet-50 to 558 patients (351 training, 109 internal validation, 2 external cohorts) for PDAC vs. CP classification, achieving AUCs of 0.953–0.986 and enhancing radiologists’ sensitivity in independent reader tests. Finally, Liang et al. (2022) [66] examined 193 pancreatic cystic neoplasm (PCN) cases—serous (*n* = 99), mucinous (*n* = 55), and intraductal papillary mucinous (*n* = 39)—extracting 1067 CECT-based radiomics features plus transfer learning-based deep features. Their fused model (radiomics + DL + clinical/morphological) reached AUC = 0.916 for SCAs and 0.973 for MCA vs. IPMN, underlining the value of integrated multi-modal analytics in preoperative PCN classification.

Table 1 includes details of all the selected works on AI-based radiomics for disease classification, including used datasets, software, extracted features, machine learning (ML) or deep learning (DL) models, and evaluation results.

### 4.2. Disease Detection

Korfiatis et al. (2023) [67] employed a modified 3D ResNet with Squeeze-and-Excitation and attention modules to detect PDAC on 696 portal-phase CTs (plus 1080 control scans) and achieved a mean accuracy of 92% (AUROC = 0.97). Their deep learning model, trained in TensorFlow on NVIDIA GPUs, demonstrated an 84% accuracy (AUROC = 0.91) on pre-diagnostic CTs acquired up to 36 months before clinical diagnosis. Yet, due to the nature of the study, a retrospective study, selection bias may have been implemented. Another limitation was that the output model presented the results intentionally in categories such as cancerous or control in order to address the need for early-stage PDA detection in asymptomatic patients. Focusing on blood-based biomarkers, Alizadeh Savareh et al. (2020) [68] analyzed 671 miRNA expression profiles from four GEO datasets (GSE113486, GSE59856, GSE85589, and GSE106817). They employed a hybrid feature selection pipeline (PSO, ANN, and NCA) and MATLAB-based neural networks, identifying a five-miRNA signature (miR-663a, miR-1469, miR-92a-2-5p, miR-125b-1-3p, and miR-532-5p) with 93% accuracy in distinguishing PC patients from healthy controls. Shifting to MRI, D’Onofrio et al. (2021) [69] examined 91 preoperative scans (1.5T Siemens and Philips systems) in IPMN patients, achieving 89.01% accuracy in detecting ≥5 mm mural nodules and finding that ADC-based entropy significantly correlated with higher tumor dysplasia.

Xia et al. (2023) [70] developed FELIX, a 3D U-Net-based framework for dual-phase CT imaging, training on 3192 scans (PDAC, PanNETs, cysts, and normal) and validating on 1846 scans from multiple institutions. FELIX reached a 97% sensitivity and 99% specificity for PDAC detection, maintaining >90% sensitivity externally. Chen et al. (2023) [71] introduced CancerUniT, a Transformer-based, multi-organ, and multi-disease model trained on 10,673 CT cases from eight major cancers plus 1055 normals. By integrating hierarchical tumor–organ learning, CancerUniT recorded a 93.3% sensitivity, 81.7% specificity, and a 4.5× speed improvement over standard methods. Finally, Zhang et al. (2020) [72] proposed a ResNet-101-based Faster R-CNN with Augmented Feature Pyramid Networks (AFPNs) and Self-Adaptive Feature Fusion for 2890 contrast-enhanced CT images from The Affiliated Hospital of Qingdao University. Their approach achieved an AUC of 0.9455, 83.76% sensitivity, and 91.79% specificity, outperforming established detection frameworks like Faster R-CNN, Mask R-CNN, and YOLO.

**Figure 4:**
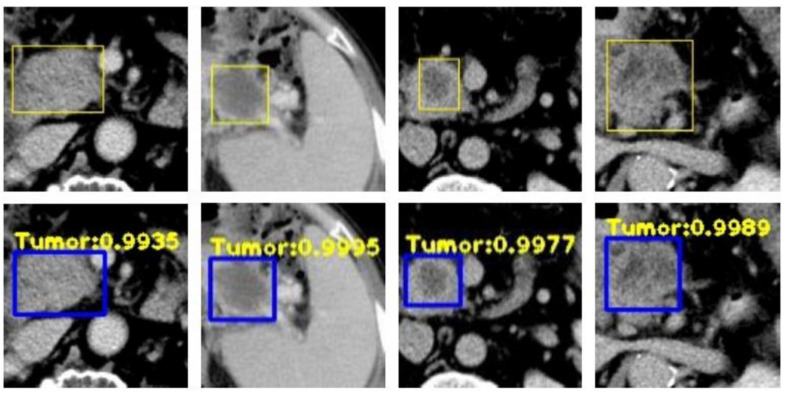
Example results of tumor detection. The first row refers to ground truth, and the second row to the corresponding detection results of the proposed method [79]. Note that numbers in the images concern detection results presented in [79], and they have no meaning at this point.

Chen et al. (2021) [73] applied a radiomics-based XGBoost classifier to 436 PDAC cases and 479 controls (National Taiwan University Hospital), plus 182 PDAC and 82 controls (TCIA, MSD) for external validation. They extracted 88 IBSI-compliant CT features using PyRadiomics and achieved a 95% accuracy on Taiwanese test data (100% specificity) and 86.5% accuracy on U.S. data. Chen et al. (2022) [74] then developed a deep learning CAD tool—encompassing a CNN-based segmentation network and an ensemble classifier—trained on 546 PC and 733 control CTs, with a nationwide validation of 1473 real-world scans. Their model attained an 89.9% sensitivity and 95.9% specificity (AUC = 0.96) internally and performed comparably (AUC = 0.95) in external datasets. However, the author reported some limitations. Firstly, radiologist reports were not available in the NHI dataset they used, so comparison between the CAD tool they proposed and the radiologists was not possible. Also, variations in imaging parameters and quality were implemented due to the NHI dataset. Chu et al. (2019) [75] explored radiomics in 190 PDAC vs. 190 healthy donor CTs, extracting 478 features and using mRMR plus a Random Forest (3000 trees) to reach a near-perfect 99.2% accuracy (AUC = 99.9%). Similarly, Liu et al. (2019) [76] employed a Faster R-CNN (with VGG16) on 4385 training and 1699 validation CT images for automatic cancer detection, yielding an AUC of 0.9632 and significant time savings compared to radiologists. Abel et al. (2021) [77] focused on detecting cystic lesions via a two-step nnU-Net in 543 cysts from 221 CTs, achieving up to 87.8% sensitivity for lesions ≥ 220 mm^3^. Ozkan et al. (2016) [78], by contrast, applied an ANN to EUS images (332 total, 202 cancer, and 130 non-cancer), extracting 122 texture-based radiomic features and reaching 87.5% accuracy overall, emphasizing age-specific training. Finally, Zhang et al. (2020) [79] targeted gene-level REOs in 573 PDAC and multiple normal/pancreatitis tissues from GEO and TCGA, narrowing over 30 million gene pairs to nine via mRMR, then training an SVM that attained 98.77% accuracy (and 100% specificity on TCGA), highlighting a robust cross-platform biomarker for early PDAC detection. Figure 4 illustrates an example of a disease detection case from Zhang et al.

Deng et al. (2021) [80] developed a radiomics-based multiparametric MRI model to distinguish PDAC from mass-forming chronic pancreatitis (MFCP) in a retrospective cohort of 119 patients (64 training, 55 validation). They extracted features from T1WI, T2WI, arterial, and portal phases with IBEX and applied LASSO + SVM, achieving AUCs of up to 0.997 in training and 0.962 in validation—significantly surpassing a clinical model’s performance. Shifting to subregional analysis, Javed et al. (2022) [81] studied 108 contrast-enhanced CT scans from healthy, pre-diagnostic, and diagnostic PDAC groups, segmenting the pancreas into head, body, and tail. Their Naïve Bayes classifier, employing RFE-based feature selection, reached 89.3% accuracy, outperforming whole-pancreas approaches. Similarly, Qureshi et al. (2022) [82] analyzed 108 pre-diagnostic CT scans (36 control, 36 pre-diagnostic, and 36 diagnostic) to train another Naïve Bayes model that attained 86% accuracy, underscoring the value of textural changes months to years before a PDAC diagnosis. However, the author stated that despite the fact that they made a thorough exploration of the datasets they used, the amount of eligible data was relatively low. This may have resulted in an overfitting problem introduced in their analysis and results.

Park et al. (2022) [83] introduced a deep learning model trained on 852 cases, with two test sets (603 and 589 patients), using a 3D nnU-Net pipeline for lesion segmentation and ensemble classification. Sensitivity reached 98–100% for solid lesions and 92–93% for ≥1 cm cystic lesions, though specificity remained somewhat lower than that of radiologists. Moving to pre-diagnostic detection, Mukherjee et al. (2022) [84] extracted 88 radiomic features from the CT scans of 155 future PDAC patients (3–36 months before diagnosis) and 265 controls. An SVM classifier delivered 92.2% accuracy (AUC = 0.98), notably surpassing radiologists (AUC = 0.66). Finally, Chen et al. (2023) [85] expanded this approach by analyzing 227 non-CP and 70 CP pre-diagnostic CTs, extracting 111 quantitative imaging features and classifying with a conditional SVM. Achieving 94–95% accuracy (AUC = 0.98–0.99) in non-CP and 100% in CP, the model retained strong predictive power even 2–3 years pre-diagnosis, showcasing the promise of radiomics-based AI for early PDAC detection.

Frøkjær et al. (2020) [86] used an MRI-based radiomics approach in a dataset of 77 chronic pancreatitis (CP) patients and 22 healthy controls, extracting 851 texture features from diffusion-weighted imaging (DWI) and training a Bayes classifier with a 10-fold cross-validation. Their model reached 98% accuracy, illustrating the potential of MRI texture analysis for CP classification. Gonoi et al. (2017) [87] retrospectively evaluated contrast-enhanced CT scans in 1848 patients (9 of whom later developed pancreatic ductal adenocarcinoma, PDAC) from a hepatocellular carcinoma (HCC) follow-up cohort, identifying subtle parenchymal changes and main pancreatic duct (MPD) irregularities up to 34 months before PDAC diagnosis. As a limitation, the authors indicate a bias that may have been injected in the study, as the cohort included only HCC patients who have received multiphase CT over a period of years, and the sample size was relatively small. Moving to deep learning, Si et al. (2021) [88] trained a fully end-to-end model—combining ResNet18 (pancreas localization), U-Net32 (segmentation), and ResNet34 (tumor classification)—on 143,945 training and 107,036 validation CT images from The First and Second Affiliated Hospitals of Zhejiang University, achieving an AUC of 0.871 for rapid tumor detection. Similarly, Ma et al. (2020) [89] constructed a convolutional neural network (CNN) using 7245 contrast-enhanced CT images (3494 PDAC vs. 3751 normal) from 412 patients, surpassing a 95% diagnostic accuracy and matching the performance of experienced gastroenterologists.

Focusing on population-level models, Hsieh et al. (2018) [90] developed both logistic regression (LR) and artificial neural network (ANN) approaches on the Taiwan National Health Insurance Research Database (NHIRD), encompassing 1.36 million type 2 diabetes mellitus (T2DM) patients (3092 with PDAC). The LR outperformed the ANN (AUROC 0.727 vs. 0.605), pointing to the strength of simpler models for large-scale risk prediction. Conversely, Muhammad et al. (2019) [91] trained an ANN with 18 health variables in a combined dataset of 800,114 participants from the National Health Interview Survey (NHIS) and the Prostate, Lung, Colorectal, and Ovarian (PLCO) Trial (898 PDAC cases), achieving an AUC of 0.85 and proposing a risk stratification framework. Finally, Boursi et al. (2017) [92] employed a logistic regression in The Health Improvement Network (THIN) database of 109,385 new-onset diabetes patients (390 PDAC diagnoses within three years), obtaining an AUC of 0.82. Collectively, these studies underscore the utility of radiomics and various machine learning or deep learning methods in facilitating early pancreatic disease detection and risk assessment.

Appelbaum et al. (2021) [93] used logistic regression (LR) and neural networks (NN) on Boston-area EHR data (1979–2017), comprising 594 PDAC cases vs. 100,787 controls, plus external validation with 408 PDAC cases and 160,185 controls. LR outperformed NN (AUC 0.71 vs. 0.68 in validation) in detecting high-risk PDAC patients up to one year before diagnosis, demonstrating a cost-effective screening tool by flagging a fraction of patients with an elevated risk. Das et al. (2008) [94], meanwhile, applied digital image analysis (DIA) and an ANN to 228 EUS image features in 110 PC, 99 CP, and 110 normal pancreas cases; after PCA feature reduction, their ANN achieved an AUC of 0.93, including 100% accuracy in differentiating CP from normal tissue. A limitation of this study was related to the cohort that consisted of digital EUS images with fixed settings in terms of gain and contrast; thus, the use and test of different settings was not feasible, possibly hiding a bias to the final diagnosis and results.

Urman et al. (2020) [95] took a multi-omic route, analyzing 129 bile samples (57 PDAC, 36 CCA, and 36 benign) with mass spectrometry and an NN model. Their final 10-lipid, 5-protein biomarker panel reached an AUC of 1.00, perfectly distinguishing PDAC from benign biliary strictures. By contrast, Liu et al. (2020) [96] trained a patch-based CNN on 370 PDAC and 320 controls, achieving near-perfect sensitivity and specificity locally, but with some performance drop (79% sensitivity, 98% specificity) on a U.S. dataset—still outperforming radiologists for sub-2 cm tumors. Finally, Săftoiu et al. (2008) [97] integrated EUS elastography with a multilayer perceptron (MLP) neural network in 68 patients (32 PC, 11 CP, 22 normal, and 3 NET), achieving up to 90% accuracy (AUC 0.965) in distinguishing PC from CP, indicating that machine learning-based elastography can be a robust tool for non-invasive pancreatic lesion characterization. Details of all aforementioned studies for disease detection are summarized in Table 2.

### 4.3. Survival Prediction

Cheng et al. (2019) [98] retrospectively analyzed 41 unresectable PDAC patients undergoing chemotherapy, extracting texture features (mean, SD, entropy, skewness, and kurtosis) from TexRAD across multiple spatial scales on pre- and post-treatment CT images. They found that a higher pre-treatment SD at SSF = 3/4 and skewness at SSF = 3 predicted longer progression-free and overall survival, while post-treatment features were less predictive. In a similar radiomics-based survival context, Khalvati et al. (2019) [99] used two cohorts (training: 30, validation: 68) of resectable PDAC patients, extracting 410 features from PyRadiomics and discarding low-reproducibility ones. Sum Entropy and Cluster Tendency (GLCM) were significantly prognostic, yielding hazard ratios up to 1.56 (*p* = 0.005) in the validation set. Yun et al. (2018) [100] assessed 88 resected pancreatic head cancer patients, measuring histogram and GLCM texture on preoperative CT. Lower contrast and standard deviation and a higher correlation (indicating homogeneous tumors) correlated with poorer DFS, highlighting texture heterogeneity as a non-invasive biomarker. However, the author reported some limitations. Due to the nature of the study, a retrospective study, a selection bias may have been introduced. Potential variables affecting tumor enhancement on the contrast-enhanced CT scans, such as cardiac output or body mass, were not taken into account. Eilaghi et al. (2017) [101] similarly extracted five GLCM-based features (uniformity, entropy, dissimilarity, correlation, and IDN) in 30 resectable PDAC cases, finding dissimilarity and IDN most predictive of overall survival (*p* < 0.05). Meanwhile, Miyata et al. (2020) [102] analyzed tumor marker indices (CA19-9, CEA, DUpan-2, and SPan-1) in 183 resected PDAC patients, showing that high Pre-TI (2-3) predicted a worse relapse-free and overall survival (HR~2.3), offering a straightforward biomarker-based prognostic tool.

**Figure 5:**
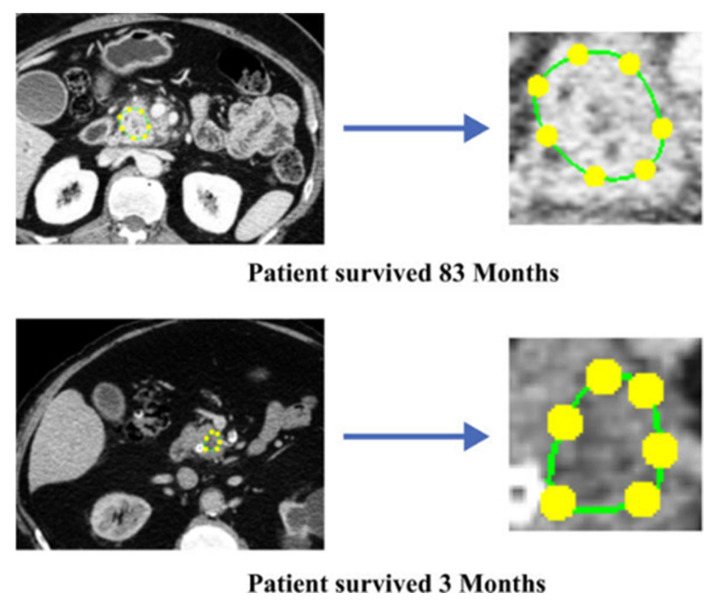
Representative images of patients with good and poor overall survival. The magnified tumor region supports the hypothesis that heterogeneously hypo-attenuating tumors are prognostic of poor survival [104].

Healy et al. (2022) [103] integrated PyRadiomics-derived features with clinical data in 352 resectable PDAC patients (training) plus 215 external validations, using a LASSO Cox model that modestly improved the C-index over clinical-only models (0.545 vs. 0.497), though performance dropped externally. In a similar quantitative CT approach, Attiyeh et al. (2018) [104] evaluated 161 chemotherapy-naive PDAC cases, extracting texture metrics in MATLAB and building two Cox models (Model A with CA19-9 + radiomics, Model B with additional pathological data). Model A attained a C-index of 0.69 and improved to 0.74 in Model B. Figure 5 illustrates representative images of patients with good and poor overall survival.

Xie et al. (2020) [105] likewise developed a radiomics nomogram (300 extracted features reduced by LASSO) in 220 resectable PDAC patients (147 training, 73 validation), achieving C-indexes up to 0.762 for DFS. Turning to the neoadjuvant setting, Kim et al. (2019) [106] studied 45 PDAC patients who received CCRT or chemotherapy pre-surgery, extracting texture changes (entropy, GLCM entropy) from pre- and post-therapy CT. Higher subtracted entropy predicted longer overall survival (HR = 0.159, *p* = 0.005). Finally, Choi et al. (2019) [107] examined 66 resected PDACs on a T2-weighted MRI using TexRAD for the mean, SD, entropy, skewness, and kurtosis, with a higher entropy (SSF4) associated with worse overall survival (HR = 4.347, *p* = 0.002). None of these studies employed deep learning or advanced ML pipelines; all relied on traditional radiomics/statistical modeling to highlight texture-derived prognostic biomarkers for PDAC therapy planning.

Parr et al. (2020) [108] analyzed 74 pancreatic cancer patients with pre-SBRT CT scans, extracting over 800 radiomic features via 3D Slicer. A 6-feature radiomic signature for overall survival (OS) yielded a C-index = 0.66 (vs. 0.54 for clinical models), while a 7-feature signature improved local–regional recurrence prediction (AUC = 0.78 vs. 0.66 clinically). Combining radiomic and clinical variables further improved performance (C-index = 0.68 for OS). Cozzi et al. (2019) [109] similarly investigated 100 SBRT patients, using LifeX-extracted CT features in a multivariate Cox model. Their clinical–radiomic signature significantly predicted OS (C-index = 0.73–0.75) and local control (0.69–0.75), stratifying patients into risk groups (median OS 9.0 vs. 14.4 months). Both studies relied on standard radiomics and statistical modeling.

Focusing on multiparametric MRI, Tang et al. (2019) [110] assessed early recurrence (≤12 months) in 303 resectable pancreatic cancers. Manual segmentations on T1-, T2-, and contrast-enhanced MRI yielded 328 radiomic features reduced by LASSO; their radiomic nomogram outperformed clinical models (AUC = 0.87–0.88). Limitations were related to the nature of the study, a retrospective study, possibly introducing a selection bias. Moreover, the used sample size was relatively small. Finally, the diffusion-weighted MR images with different b values were abandoned over a long time period because of poor consistency between the two hospitals. Wang et al. (2022) [111] then built a CT-based radiomics–clinical nomogram for 184 resectable PDACs (111 training, 28 internal, and 45 external validations), extracting 1409 PyRadiomics features and integrating CA19-9. This combined model surpassed TNM staging (C-index = 0.713 vs. 0.616). Chakraborty et al. (2017) [112] examined 35 patients’ pre-treatment CT scans, extracting 255 texture features and using a fuzzy mRMR plus naïve Bayes pipeline; the best performance reached AUC = 0.90 (82.86% accuracy) via leave-one-image-out validation.

Meanwhile, Kaissis et al. (2019) [113] used DWI-based ADC maps in 132 PDAC patients (102 training, 30 validation) with a Random Forest on selected PyRadiomics features (recursive elimination), reaching AUC = 0.90 in predicting above/below-median OS. In another CNN approach, Zhang et al. (2020) [114] employed a 6-layer deep network trained on 98 PDAC CT scans (68 training, 30 validation), achieving a C-index = 0.651 (vs. 0.603 for transfer learning-based Cox, 0.491 for radiomics-based Cox). Shi et al. (2021) [115] integrated CT radiomics (LASSO-derived), CA19-9, the skeletal muscle index, grade, and chemotherapy status in 299 resectable PDAC patients (210 training, 89 validation). Their combined Cox model delivered a C-index = 0.74, surpassing clinical-only (0.68) or TNM staging (0.59). Finally, Rezaee et al. (2016) [116], without any radiomics or AI, tracked 616 IPMN resections at Johns Hopkins, noting that high-grade dysplasia significantly increases PDAC risk despite not being itself malignant, emphasizing the need for vigilant surveillance. Table 3 includes details of all reviewed works regarding survival prediction.

### 4.4. Treatment Response

**Figure 6:**
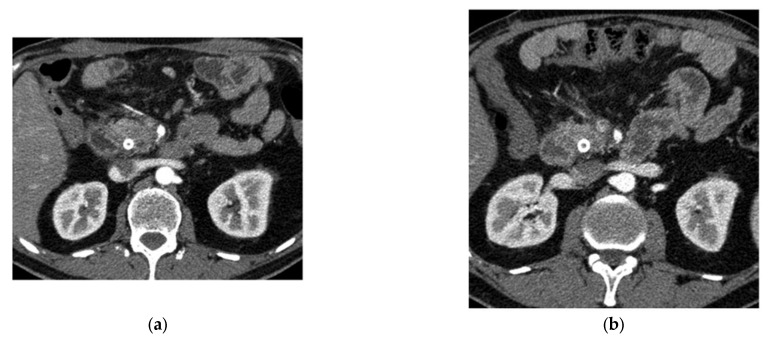
CT study of pancreatic adenocarcinoma before and after chemotherapy: (**a**) CT study of locally advanced ductal adenocarcinoma of the pancreatic head; (**b**) CT control after chemotherapy [118].

Abraham et al. (2021) [117] integrated clinical and next-generation sequencing data from real-world and the TRIBE2 trial to create a 67-gene FOLFOXai signature for metastatic colorectal cancer patients. Trained on time-to-next treatment (TTNT) and validated on PFS and OS, it showed a significant predictive advantage for oxaliplatin-based (FOLFOX/FOLFOXIRI) regimens in multiple cohorts, extending to esophageal/gastroesophageal junction cancers and PDAC. In contrast, Ciaravino et al. (2018) [118] focused on CT texture analysis in PDAC patients (*n* = 17), downstaged to the resectable status post-chemotherapy. Using MaZda software, changes in kurtosis distinguished responders from those with disease progression, underscoring CTTA’s potential to track a neoadjuvant response. Figure 6 illustrates indicative CT scans of PDAC before and after chemotherapy.

Turning to surgical risk, Mu et al. (2020) [119] applied a convolutional neural network to preoperative CE-CTs of 513 pancreatoduodenectomy (PD) candidates, producing a deep learning score outperforming the traditional Fistula Risk Score in predicting clinically relevant postoperative pancreatic fistulas (AUC = 0.85 vs. 0.78). Nasief et al. (2019) [120] then introduced a machine learning delta-radiomics approach with Bayesian neural networks on 2520 daily CTs from 90 patients during CRT, achieving an AUC = 0.94 for early treatment response prediction. A subsequent study by Nasief et al. (2020) [121] combined these delta-radiomics features with CA19-9 levels in 24 patients, enhancing the survival prediction (C-index rising from 0.57 to 0.87).

Focusing on neoadjuvant therapy, McClaine et al. (2010) [122] reviewed 29 borderline resectable PDAC cases, concluding that gemcitabine-based regimens improved resection rates (46%) and median survival (23.3 vs. 15.5 months). Yue et al. (2017) [123] leveraged pre-/post-RT PET/CT in 26 patients (various RT regimens) and found changes in texture features like homogeneity/variance correlated with survival. The authors reported that the small cohort size was a major limitation of the study. Meanwhile, Cassinotto et al. (2013) [124] showed that neoadjuvant therapy reduced the specificity of MDCT-based resectability prediction (58% vs. 83%) by overestimating the tumor size and vascular invasion.

Chen et al. (2017) [125] explored daily diagnostic CTs in 20 CRT patients for a radiomic assessment of the treatment response, noting significant changes in the mean CT number and skewness after two weeks. Rigiroli et al. (2021) [126] aimed to detect the SMA involvement on CT in 194 PDAC cases (148 post-neoadjuvant), extracting 1695 features and finding a final five-feature logistic model (AUC = 0.71), surpassing radiologists’ AUC = 0.54. Along similar lines, Bian et al. (2020) [127] used 1029 portal-phase radiomic features in 181 pancreatic head cancer cases to predict the SMV resection margin, achieving an AUC = 0.75. Finally, Gregucci et al. (2022) [128] built a radiomics-based logistic model on 37 LAPC patients before SBRT, identifying the GLCM25_Correlation and NID25_Busyness as key predictors of a local response (AUC = 0.851), suggesting a way to tailor dose strategies for improved outcomes. Table 4 summarizes details regarding selected works on treatment response.

### 4.5. Radiogenomics

**Figure 7:**
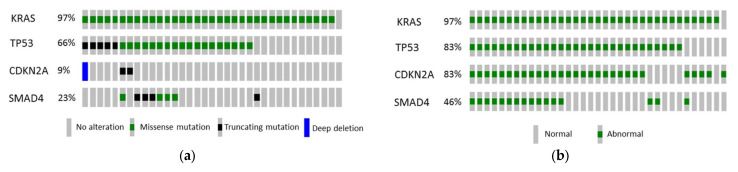
Oncoprint taken from the work of Attiyeh et al. [130] showing (**a**) genomic alterations; (**b**) status as determined by genomic alterations and IHC. Columns represent patients in the cohort (*n* = 35).

McGovern et al. (2018) [129] analyzed 121 PanNET patients on multiphasic CE-CT, noting that ALT-positive tumors were linked to a lobulated shape (*p* = 0.001), necrosis (*p* = 0.002), vascular invasion (*p* < 0.001), duct dilatation (*p* < 0.001), and hepatic metastases (*p* < 0.001). Although a multivariate model (including duct dilatation, hepatic metastasis, and size ≥ 3 cm) only reached an AUC of 0.58, the study highlighted that intratumoral calcifications and metastatic burden predict worse survival regardless of ALT status. Yet, the relatively small sample size may have limited the predictive value of specific CT characteristics. Also, CT technique was not uniform, as 10 studies were performed at external institutions, and the remaining 111 studies were performed inside the institution the authors worked in. Shifting to PDAC radiogenomics, Attiyeh et al. (2019) [130] examined 35 resected cases with targeted sequencing (KRAS, TP53, CDKN2A, and SMAD4) and 255 CT radiomics features (GLCM, RLM, LBP, FD, etc.), finding 28 and 32 features predictive of SMAD4 and TP53 mutations, respectively. The number of mutated genes correlated with a shorter survival (*p* = 0.016), and stromal content estimation (R^2^ = 0.731) underscored the value of imaging in characterizing the genotype and microenvironment. Figure 7 illustrates oncoprints showing genomic alterations from the work of Attiyeh et al. [130].

Lim et al. (2020) [131] then used 18F-FDG PET/CT in 48 PDACs, extracting 35 features with CGITA, and showed KRAS mutations aligned with low-intensity texture measures (AUC up to 0.829), while SMAD4 mutations were associated with SUV skewness (AUC = 0.727) and short-run/high-intensity emphases. Reported limitations included the retrospective design and the small sample size that may have affected the results of the study. Also, several technical limitations in the method of PET-based radiomics may have occurred, since textural feature calculations are affected by many factors such as the signal to noise ratio of volume of interest definition. Similarly, Iwatate et al. (2020) [132] applied PyRadiomics to 2074 early-/late-phase CT features in 107 PDACs; their XGBoost model reached AUC = 0.795 for p53 and 0.683 for PD-L1, with the p53 status strongly predicting worse survival (*p* = 0.015). In a larger ultrasound-based approach, Tang et al. (2024) [133] evaluated 151 in-house plus 54 CPTAC-PDAC patients and tested 77 ML combinations on 1239 radiomics features, achieving AUCs of 0.84–0.85 for lymph node metastasis; subsequent WGCNA analysis linked key radiomic markers to proliferation pathways. Hinzpeter et al. (2022) [134] also investigated the mutation status (KRAS, TP53, SMAD4, and CDKN2A) in 47 PDACs (portal-phase CT, LIFEx), identifying HU_Skewness, GLZLM_SZLGE and NGLDM_Coarseness as top predictors (Youden indices up to 0.67). Finally, Iwatate et al. (2022) [135] assessed ITGAV expression in 107 PDACs (3748 CT features) via XGBoost, finding predicted high integrin αV levels (AUC = 0.697) associated with a significantly worse OS (*p* = 0.048) and suggesting that radiogenomics can non-invasively profile pivotal molecular targets for precision PDAC management. Table 5 summarizes details from the selected literature on radiogenomics.

### 4.6. Deep Radiomics Fusion Models

Dmitriev et al. (2017) [136] proposed a hybrid radiomics + deep learning model for pancreatic cyst classification, applying a Random Forest (RF) with 14 quantitative radiomic features (patient demographics, lesion shape, and intensities) alongside a 2D CNN for higher-level feature extraction in a contrast-enhanced CT. They fused both models via the Bayesian ensemble, achieving an overall accuracy of 83.6% across 134 patients with IPMNs, MCNs, SCAs, and SPNs. Notably, the RF excelled at smaller cysts, while the CNN better characterized larger lesions. The authors emphasized that further work should target earlier malignancy detection and multi-institutional validation.

Ziegelmayer et al. (2020) [137] similarly integrated handcrafted radiomics from PyRadiomics (1411 features) and VGG19-based deep features (256) to differentiate PDAC from autoimmune pancreatitis (AIP) in 86 portal-phase CTs. After segmentation and feature selection, deep features yielded an AUC = 0.90 (89% sensitivity, 83% specificity), outperforming radiomics alone (AUC = 0.80). Although they operated on each feature set separately rather than fusing them, CNN-derived activations captured more nuanced patterns than traditional radiomics, highlighting the benefit of deep learning in challenging differential diagnoses.

Zhang et al. (2021) [138] explored a fusion approach for PDAC survival prediction in 98 contrast-enhanced CTs (68 training, 30 validation). They extracted 1428 handcrafted features (PyRadiomics) plus 35 transfer learning-based deep features (LungTrans CNN). Testing multiple fusion schemes (PCA, Boruta, Cox, and LASSO), they ultimately introduced a novel risk score method combining radiomics and deep feature Random Forests, achieving an AUC of 0.84–notably better than traditional methods. However, interpretation and multi-site validation remain key next steps.

Wei et al. (2023) [139] took a multi-modal path, fusing radiomics and deep learning features from 18F-FDG PET/CT to distinguish PDAC (*n* = 64) from AIP (*n* = 48). Radiomics captured histogram, texture, and morphology from both PET and CT, while VGG11 CNN extracted high-level features. Their multidomain fusion model (MF_model) reached an AUC = 96.4%, outperforming the radiomics-only (89.5%) and deep-only (93.6%) approaches. The authors noted that a larger, externally validated dataset will be essential to confirm these strong results. It should be noted that the authors pointed out two major limitations in their work; the sample size was relatively small, so a 5-fold cross-validation was used to reduce the risk of model overfitting, while external validation of the model was not performed with an external dataset.

Yao et al. (2023) [140] applied a multi-institutional MRI pipeline for intraductal papillary mucinous neoplasm (IPMN) risk stratification, assembling 246 T1-/T2-weighted MRI scans from five centers. After nnUNet-based pancreas segmentation, 107 radiomics features were extracted and combined with deep features from five CNN architectures (DenseNet, ResNet18, AlexNet, MobileNet, and ViT) plus clinical factors. Their weighted-averaging fusion significantly boosted accuracy from 61.3 to 71.6% (single CNN/radiomics) to 81.9%. Limitations included cross-center intensity variability; future studies aim to integrate molecular markers and expand prospective validation.

Finally, Vétil et al. (2023) [141] introduced a mutual-information-minimized (MI) fusion approach for early PDAC detection using 2319 training and 1094 test CT scans. Handcrafted radiomics (PyRadiomics) were paired with deep features from a VAE, but critically, the VAE was trained to minimize redundant information relative to the handcrafted feature space. This MI-based technique boosted AUC by ~1.13% over handcrafted alone, indicating that ensuring deep features truly complement radiomics can improve classifier performance. Further improvements in feature interpretability and unified modeling were highlighted as the next steps, especially for large-scale clinical adoption. Details about the selected literature focused on fusion models are summarized in Table 6.

## 5. Datasets, Features, and Methods

A wide range of both public and private datasets underpins the development of radiomics and AI techniques for pancreatic cancer, encompassing openly available resources like TCIA, MSD, NIH-Pancreas CT, and GEO profiles, as well as extensive in-house clinical cohorts from various institutions worldwide.

In parallel, the extracted features commonly include shape descriptors, first-order intensities, and texture metrics (frequently derived from GLCM, GLRLM, or wavelet transformations), while deep neural networks provide high-level embeddings that capture nuanced morphological cues. These approaches—often combined with feature selection strategies such as LASSO or mRMR—have consistently driven a strong performance in tasks such as disease detection, lesion classification, survival prediction, and treatment response assessment.

### 5.1. Datasets

Across the 126 reviewed studies, a variety of datasets were used, ranging from fully public repositories to strictly in-house patient cohorts. Several investigations relied on large, well-known public data resources such as The Cancer Imaging Archive’s TCIA-CPTAC collection, the Medical Segmentation Decathlon (MSD), NIH-Pancreas CT scans, GEO profiles, and population databases like NHIS, THIN, LHDB, or CBioPortal. In these instances, images or molecular data are openly available, allowing other researchers to replicate or extend the findings. Studies that combined multiple sources often leveraged a public dataset for external validation while training primarily on local or in-house cases.

A substantial number of investigations, however, used private institutional data without releasing them publicly—usually because the imaging or clinical records could not be shared due to privacy or regulatory constraints. In many of these, the institutions were explicitly named (for example, Johns Hopkins Hospital, National Taiwan University Hospital, or Mayo Clinic), underscoring a “private but institution-named” type of data usage. In other cases, the data source was described only as “in-house,” “local,” or “private,” with no specific hospital or facility identification.

When categorizing all 126 studies by data type, only 12 studies made use of exclusively public datasets, often involving well-established collections like TCIA, MSD, NIH-Pancreas, GEO, or broad population databases (NHIS, THIN, CBioPortal, and LHDB). More than half of the studies drew on private data while explicitly mentioning the originating institution (e.g., a specific university hospital or medical center), reflecting collaborations and retrospective reviews of patient cohorts. The remaining subset relied on non-public, in-house datasets without naming the hospital or clinical setting, typically referring to them in generic terms such as “local,” “private,” or “institutional” data. Overall, the majority were private (with or without a named institution), whereas roughly one in eight leaned solely on public repositories, illustrating both the opportunities and constraints researchers face in accessing large-scale pancreatic imaging cohorts. Table 7 includes all public datasets used in each clinical application defined in this work.

**Figure 8:**
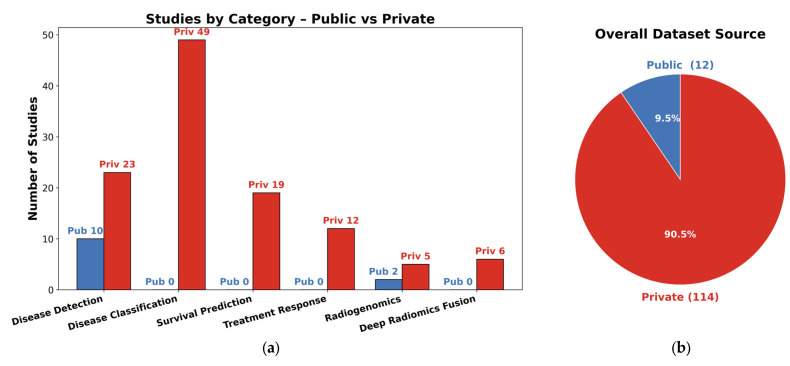
Graphical illustrations of (**a**) public and private datasets per clinical application; (**b**) percentage of public vs. private datasets from overall dataset sources.

In order to provide a more complete picture of the used datasets in the selected literature, in Table 8, the overall used datasets per clinical application are summarized. Figure 8 graphically illustrates the percentage of public versus private datasets, revealing a total of 12 public (9.5%) vs. 114 private (90.5%) datasets.

### 5.2. Features and Methods

The literature on pancreatic imaging has increasingly integrated advanced computational techniques, combining both handcrafted radiomics and deep learning approaches. Researchers extract hundreds to thousands of features—ranging from shape and intensity descriptors to complex texture metrics (such as those derived from the Gray-Level Co-Occurrence Matrix and wavelet transformations)—which are then distilled using feature selection methods like LASSO or mRMR. Concurrently, deep convolutional neural networks (CNNs) capture abstract, high-level representations from imaging data, with architectures often augmented by attention mechanisms or fusion strategies. Together, these methodologies not only enhance tumor detection and classification but also improve prognostic predictions and treatment response assessments, demonstrating the potential for more accurate, non-invasive clinical decision support in pancreatic cancer.

In pancreatic cancer detection, researchers have developed models that harness both handcrafted radiomic features and deep learning methods from CT and MRI scans. Radiomics approaches typically extract hundreds of features—such as shape descriptors, first-order intensity metrics, and texture measures (often derived from matrices like GLCM, GLRLM, GLSZM, and NGTDM)—using standardized protocols and feature selection methods like LASSO, mRMR, or PCA. In parallel, deep learning methods employ 2D or 3D convolutional neural networks (e.g., modified ResNet, U-Net, and Faster R-CNN), often pre-trained on large image datasets to automatically extract high-level features that capture subtle morphological and textural details. Many studies report a robust performance with AUCs above 0.90, underscoring the effectiveness of these techniques—especially the frequent use of GLCM-based features and attention mechanisms—to accurately distinguish pancreatic cancer from benign conditions and healthy tissue.

For classifying various pancreatic pathologies—including PDAC, autoimmune pancreatitis, chronic pancreatitis, PanNETs, and cystic neoplasms—researchers have employed both handcrafted radiomics and deep learning methods. Handcrafted approaches typically extract hundreds of features, which are then reduced via feature selection to a subset of 10–40 key descriptors such as GLCM correlation, entropy, and run-length measures, alongside morphological features like tumor volume and shape. Deep learning techniques, using patch-level or fully 3D CNNs, further enhance segmentation and subtype classification, often with ensemble methods boosting overall performance. Integrating radiomic features with deep learning embeddings or clinical variables (e.g., CA19-9, CEA) consistently yields high accuracy, with many studies reporting AUC values between 0.80 and 0.95.

Survival prediction studies typically rely on radiomic features extracted from preoperative CT or MRI scans, often combined with clinical biomarkers (e.g., CA19-9, CEA, and tumor grade) or body composition metrics like the skeletal muscle index. Researchers usually extract hundreds to thousands of features—including first-order statistics, texture measures (such as those from GLCM and GLRLM), and morphological descriptors—and then reduce these features using methods like LASSO or the univariate Cox analysis. These refined features are integrated into multivariable survival models, most commonly using the Cox proportional hazards framework, though some studies have experimented with Random Forest survival or neural network-based models. A consistent finding is that greater tumor heterogeneity—often indicated by higher entropy or dissimilarity—correlates with poorer overall survival, and incorporating changes in these features (delta-radiomics) can further improve prognostic accuracy. Overall, combining imaging biomarkers with clinical data generally yields a superior survival prediction performance, with concordance indices frequently exceeding 0.70.

For treatment response, researchers analyze changes in radiomic and deep learning features during therapies such as neoadjuvant chemotherapy, chemoradiation, or SBRT. By tracking delta-radiomics metrics from daily or weekly CT scans, they observe longitudinal changes in texture features—such as kurtosis, skewness, and entropy—that can signal early treatment response. Deep learning segmentation networks further assist by monitoring morphological changes in tumor volume and density. Notably, combining these imaging biomarkers with clinical indicators, like CA19-9 decline, often enhances prediction accuracy, with consistent findings that increased post-therapy entropy or irregular texture is associated with resistance or an incomplete response.

Radiogenomics studies connect imaging features to genetic and molecular profiles in pancreatic cancer. Researchers extract extensive sets of radiomic features (often over 2000) from contrast-enhanced CT, MRI, or PET/CT images and correlate them with sequencing data for key mutations (e.g., KRAS, TP53, SMAD4, and CDKN2A) or gene-expression markers (e.g., integrin αV, p53 status, and PD-L1). Using feature selection methods like recursive elimination and Random Forest rankings, these studies often identify texture features—such as GLCM entropy and coarseness—as markers of high-risk molecular profiles. Additionally, some deep learning pipelines have shown that CNN-derived embeddings can track specific mutation subtypes, suggesting that non-invasive imaging biomarkers may partially predict molecular phenotypes and eventually guide targeted therapies.

Deep radiomics fusion models combine handcrafted radiomic features (such as shape, texture, and intensity-based descriptors) with high-dimensional deep learning features extracted from CNNs. By integrating these complementary data sources using ensemble techniques like Random Forests, gradient boosting, or logistic regression, these models consistently achieve 3–5% gains in accuracy or AUC over single-method approaches. Some studies even use mutual information minimization to ensure that deep features add unique value beyond traditional radiomics. Overall, this integrated approach enhances the early detection of PDAC, differentiates benign from malignant cysts, and improves survival prediction when sufficient data and careful feature engineering are available.

A number of common themes emerge across the selected literature, with a widespread reliance on GLCM-based texture metrics—such as correlation, entropy, and homogeneity—that consistently rank among the top-performing radiomics features across detection, classification, and prognostic tasks. Despite the initial extraction of hundreds or even thousands of features from imaging data, rigorous feature reduction methods, including LASSO, minimum redundancy maximum relevance (mRMR), and Random Forest ranking, typically reduce these to a focused set of fewer than 10–40 high-impact features that drive model performance. Deep learning methods, particularly those that extract CNN embeddings, complement these handcrafted features by capturing nuanced morphological cues and abstract patterns that are often missed by traditional techniques, thereby enhancing the predictive power when fused with radiomics. Moreover, key clinical biomarkers such as CA19-9 frequently appear in these models, providing valuable complementary information for treatment response and survival prediction, while additional markers like CEA or diabetic status are occasionally integrated to further refine the predictions. Although most models report accuracies or AUCs above 0.80, indicating a strong potential, variability in CT or MRI acquisition protocols can affect generalizability, which underscores the critical need for standardized imaging protocols in order to achieve consistent and reliable results across studies.

In Table 8, the feature sets and main objectives across various pancreatic imaging studies are gathered.

The literature reveals that specific radiomic features consistently serve as robust markers across multiple tasks in pancreatic imaging. Handcrafted texture metrics derived from the GLCM—particularly entropy, correlation, and homogeneity—frequently emerge as strong predictors for both detection and prognosis. In addition, wavelet-transformed features are routinely found among the top-performing subsets for classifying various pancreatic conditions, while shape features like sphericity and elongation are crucial for differentiating cystic from solid lesions and monitoring morphological changes during treatment. Deep CNN features add another layer of detail by capturing abstract patterns related to tumor boundaries and texture complexity, effectively complementing the traditional radiomics set.

Moreover, fusion models that integrate both handcrafted radiomics and deep learning embeddings consistently demonstrate incremental gains in performance, underscoring the complementary nature of these approaches. By combining imaging biomarkers with clinical variables such as CA19-9, these models achieve superior accuracy in detecting, classifying, and prognosticating pancreatic diseases. Overall, the integration of traditional feature extraction methods with data-driven deep learning techniques offers a comprehensive and robust framework for improving clinical decision-making in pancreatic imaging.

Overall, the reviewed studies highlight a consistent reliance on texture features—particularly those derived from the gray-level co-occurrence matrix (GLCM), such as entropy, contrast, correlation, and homogeneity—as well as morphological and shape descriptors including volume, surface area, compactness, and sphericity. These features were repeatedly identified as among the most discriminative across a range of clinical tasks, from disease detection and classification to survival prediction and treatment response assessment. Texture features were particularly valuable in capturing intratumoral heterogeneity, which is a key imaging correlate of tumor aggressiveness, while shape features helped quantify tumor invasiveness and spatial characteristics. Despite their frequent use, there remains considerable variation in how radiomic features are extracted and selected across studies. Differences in image acquisition protocols, segmentation methods, image preprocessing (e.g., resampling, intensity normalization), and feature definitions (e.g., IBSI-compliant vs. non-standard metrics) all contribute to variability in reported results. Feature selection strategies also differ widely—ranging from univariate statistical filtering to complex machine learning-based ranking—resulting in a limited overlap of retained features across studies, even when similar clinical endpoints are examined. This inconsistency underscores the urgent need for harmonized radiomics workflows, standardized feature definitions, and external validation on multi-institutional datasets to ensure the reproducibility and clinical credibility of radiomic biomarkers in PDAC. Without such standardization, the development of reliable, generalizable radiomic signatures for clinical use remains challenging.

**Figure 9:**
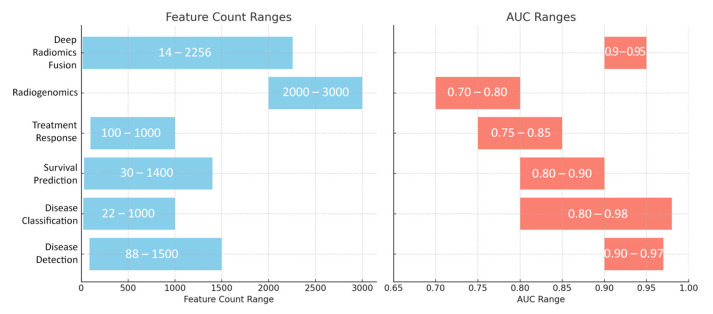
Dual-panel horizontal range bar chart illustrating feature count ranges and AUC ranges per clinical application.

In Figure 9, the horizontal range bar chart shows the approximate feature count ranges and AUC performance ranges across different clinical applications in pancreatic cancer. Each bar represents the minimum-to-maximum values reported in the selected literature for a given task category.

In order to understand not only the methodological diversity but also the practical reproducibility of AI and radiomics studies in PDAC, we catalog and analyze the software platforms, standalone tools, and programming environments reported across the literature. For this scope, the column “Software/Tool/Programming Language” exists in each table, aiming to provide readers with a transparent overview of the technical ecosystem used across the literature for different clinical applications and the deep radiomics fusion methodology. Specifically, this column helps identify which software tools (e.g., ITK-SNAP, 3D Slicer), radiomic feature extraction platforms (e.g., PyRadiomics, MaZda), and programming environments (e.g., MATLAB, Python, and TensorFlow) are most commonly employed in various stages of the AI pipeline, ranging from image segmentation and preprocessing to feature extraction and model implementation. By capturing every reported segmentation package (e.g., ITK-SNAP, 3D Slicer, and nnUNet), feature extraction framework (e.g., PyRadiomics, MaZda, and LIFEx), and modeling library or language (e.g., TensorFlow, MATLAB, R, and scikit-learn), we aim to present prevailing trends in technology adoption, highlight emerging standards, and expose gaps in the reporting literature. Specifically, the inclusion of programming languages serves to provide insight into the computational environments most commonly adopted in the reviewed literature, since programming language often reflects the underlying technological framework, community support, and evolution of development practices in the field. For example, the frequent use of Python, especially in conjunction with libraries like PyRadiomics or TensorFlow, reflects the current trend toward open-source, flexible, and well-supported development environments in radiomics and deep learning research. Meanwhile, the continued presence of MATLAB indicates its longstanding role in image processing despite being a more traditional proprietary environment. Tracking the adoption of these programming languages allows readers to understand the technological shifts in the field, anticipate tool compatibility, and assess the reproducibility and accessibility of published pipelines, especially for researchers aiming to replicate or build upon prior work. In this way, the programming language information complements the software/tool listings and offers broader context about the ecosystem surrounding radiomics research.

To this end, this tool-level survey serves three key purposes: (1) It reveals which systems researchers most often rely on for delineating tumor boundaries, computing radiomic features, and building predictive models; (2) it contextualizes shifts in the field—such as the movement from proprietary environments toward open-source, Python-based ecosystems; and (3) it identifies opaque or under reported workflows that may hinder reproducibility and impede external validation. Therefore, these insights provide a concrete roadmap for future authors towards documenting their computational pipelines with greater clarity, and for the community to coalesce around interoperable, well-supported tools that accelerate the translation of radiomics-driven AI into clinical practice.

**Figure 10:**
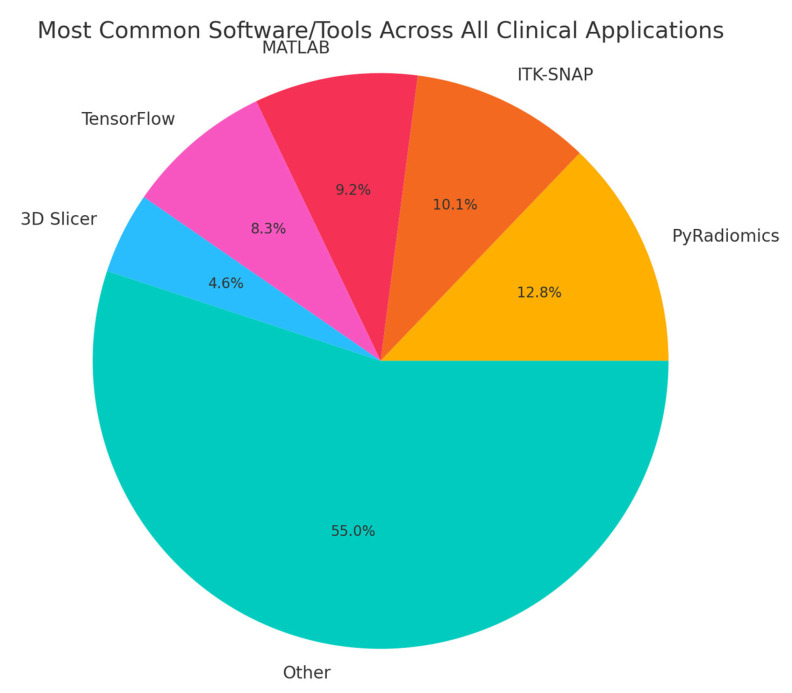
General distribution of the most used software/tools.

Regarding software and tools, the distribution of software usage is far from uniform across all reviewed studies. With almost one out of every eight studies citing it, PyRadiomics is by far the most widely used platform. This dominance is a result of its open-source nature, rigorous adherence to IBSI feature definitions, and smooth integration with machine learning workflows based on Python. A second tier of popularity is formed by ITK-SNAP (~10%) and MATLAB (~9%); the former is preferred for its easy-to-use, semi-automated segmentation features that simplify lesion delineation before feature extraction, while the latter is still widely used in academic imaging labs because of its sophisticated image processing toolboxes and extensive institutional licenses. TensorFlow (approximately 8%), which most frequently supports CNN-based feature learning or complete classification pipelines that supplement manually created descriptors, represents the gradual infiltration of deep learning frameworks into PDAC radiomics. About 5% of papers use 3D Slicer, which offers a flexible, GUI-driven solution for teams that prefer a point-and-click setting. It also benefits from community extensions like SlicerRadiomics. However, despite these clear leaders, the majority of studies fall into the “Other” category, which includes proprietary vendor software, one-off institutional tools, and, most alarmingly, papers that make no reference to their computational environment at all. This widespread lack of transparency continues to be a major limitation in the current body of PDAC research, impeding direct replication, cross-study benchmarking, and ultimately undermining the explainability and reproducibility of suggested radiomic signatures. Figure 10 illustrates the general distribution of the most commonly used software/tools across all clinical applications.

**Figure 11:**
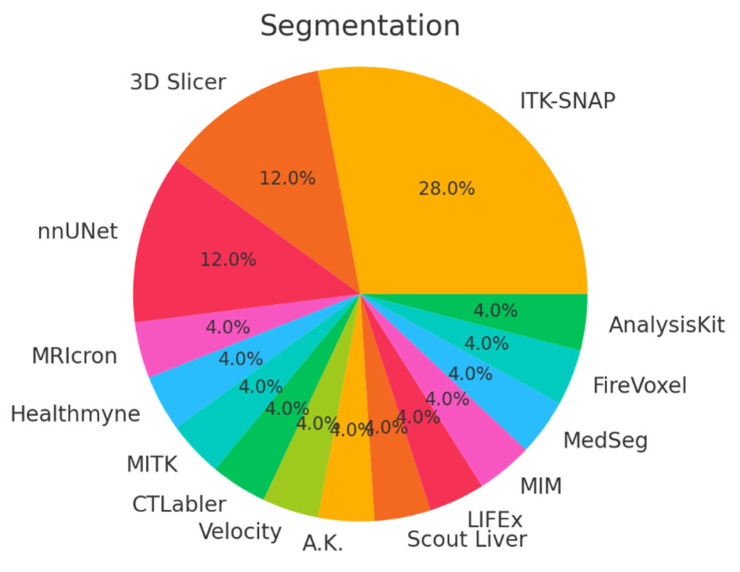
Distribution of the most used software/tools for segmentation purposes.

**Figure 12:**
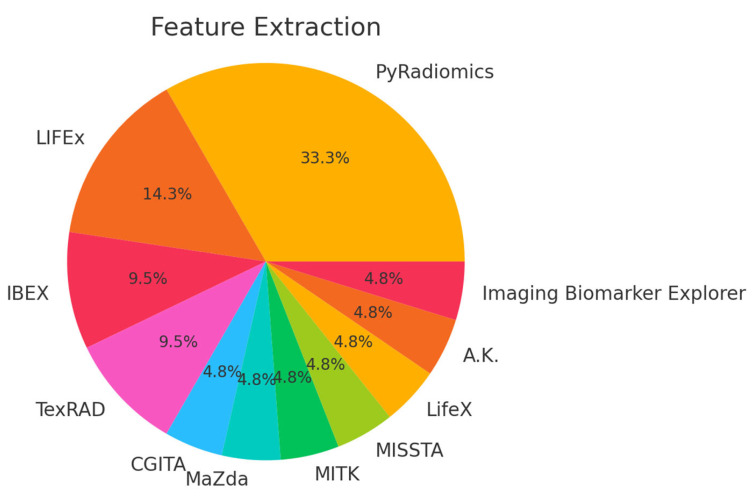
Distribution of the most used software/tools for feature extraction purposes.

**Figure 13:**
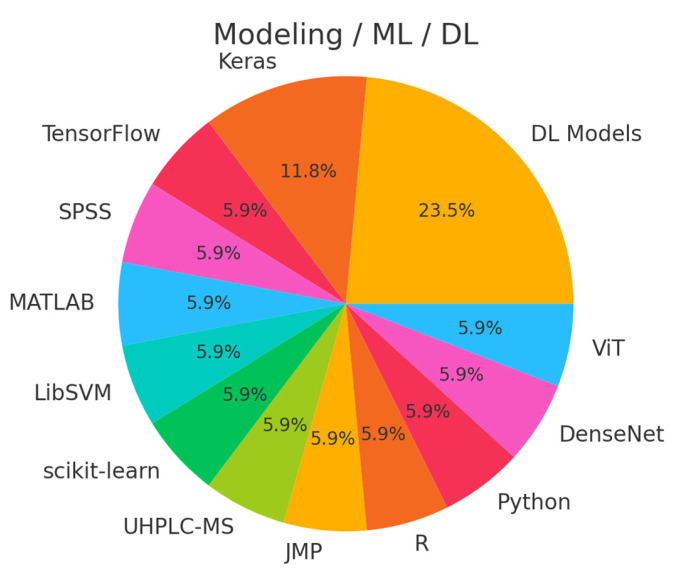
Distribution of the most used software/tools for Modeling/ML/DL purposes.

By breaking down Figure 10 by purpose, three additional figures (Figure 11, Figure 12 and Figure 13) are extracted, aiming to provide an overview of the software/tools used for feature extraction, ML/DL, and segmentation. Figure 11 illustrates the percentage of software and tools used for segmentation purposes. The segmentation pie chart reveals that ITK-SNAP is the predominant tool, used in 28% of studies for manual or semi-automated ROI delineation, likely reflecting its robust 3D brush and thresholding features. The nnUNet and 3D Slicer each account for 12%, indicating a growing adoption of deep learning-based auto-segmentation and an extensible GUI platform with numerous community plugins. The remaining 48% is split among over a dozen niche packages, such as CTLabler, Scout Liver, Healthmyne, MITK, MedSeg, and A.K. (AnalysisKit), each at roughly 4%. This long tail underscores both the methodological diversity in segmentation approaches and the absence of a single, field-wide standard, which may contribute to inter-study variability in ROI definition and downstream feature consistency.

Figure 12 presents the percentage of software and tools used for feature extraction purposes. For radiomic feature computation, PyRadiomics dominates with 33% usage, reflecting its open-source, IBSI-compliant implementation and seamless Python integration. LIFEx captures the next largest slice at 14%; this standalone application offers a GUI for the rapid extraction of first-order, shape, and texture features. IBEX and TexRAD each contribute about 10%, highlighting their roles in specialized texture analyses. A series of smaller segments, MaZda, MISSTA, CGITA, LifeX, and A.K. (AnalysisKit), and in-house tools like Imaging Biomarker Explorer, round out the landscape, each appearing in 5% or fewer studies. The prominence of a handful of platforms suggests an emerging consensus on feature extraction standards, yet the diversity of lesser-used packages points to ongoing experimentation with novel metrics and proprietary workflows.

Figure 13 presents the percentage of software and tools used for Modeling/ML/DL purposes. In predictive modeling, the “DL Models” category encompassing custom CNNs, autoencoders, and transfer learning architectures, accounts for 24% of reported frameworks, highlighting the rapid shift toward end-to-end deep learning. Keras (12%) and TensorFlow (6%) are the most cited high-level DL libraries, prized for their flexibility and GPU acceleration. The remaining third of the pie is a patchwork of statistical and machine learning environments such as MATLAB, R, SPSS, JMP, scikit-learn, LibSVM, and even mass-spectrometry analyzers (UHPLC-MS), each used by about 6% of studies. This mix signals that, while deep learning is on the rise, a significant portion of research still relies on traditional ML toolkits and statistical software, often for tasks like survival analysis or classical classifier comparisons.

### 5.3. Radiomics and Deep Radiomics Comparison

The evolution from traditional radiomics to deep radiomics marks a significant shift in how imaging features are extracted, represented, and modeled in medical image analysis. Both approaches have been widely applied in pancreatic ductal adenocarcinoma (PDAC) research, but they differ substantially in methodology, interpretability, and computational demands. This section presents a comparative discussion of traditional and deep radiomics pipelines, followed by a graphical and tabulated summary highlighting their key differences and areas of synergy.

Traditional radiomics follow a well-defined, multi-step pipeline that typically includes image acquisition, manual or semi-automated tumor segmentation, image preprocessing (e.g., resampling, normalization), handcrafted feature extraction, feature selection, and model development. The extracted features are mathematically defined and include first-order statistics (e.g., mean, skewness), texture features (e.g., GLCM-based entropy, contrast, and homogeneity), shape descriptors (e.g., volume, compactness), and higher-order transformations such as wavelets. This pipeline emphasizes interpretability and reproducibility, especially when tools such as PyRadiomics and standardized feature definitions (e.g., IBSI) are used.

Deep radiomics, on the other hand, incorporate deep learning, typically CNNs, to learn hierarchical, abstract features directly from the image data. These deep features are not predefined but are learned from training data through backpropagation, often from intermediate CNN layers. The deep radiomics workflow may still involve segmentation and preprocessing, but often uses automated segmentation (e.g., nnU-Net), data augmentation, and end-to-end modeling. In fusion models, handcrafted features are combined with deep features through concatenation or attention mechanisms, providing a hybrid representation that captures both domain-specific and data-driven imaging characteristics.

The fundamental differences between traditional radiomics and deep radiomics lie in how imaging features are generated, interpreted, and applied. Traditional radiomics relies on handcrafted features that are explicitly defined using mathematical formulas, such as first-order statistics, shape descriptors, and texture measures like GLCM or wavelets, which make the extracted features inherently interpretable and easier to correlate with known clinical or pathological characteristics. In contrast, deep radiomics use CNNs to automatically learn abstract, hierarchical representations from raw imaging data without predefined formulas. While this allows deep radiomics to capture complex, non-linear patterns that may not be easily modeled with handcrafted features, it also introduces challenges in interpretability, as the resulting feature maps are often opaque and difficult to link to specific image traits. Deep radiomics typically require larger datasets to generalize effectively and often rely on high-performance computing infrastructure for model training. Traditional radiomics, by contrast, are more modular and less resource intensive, making it suitable for smaller datasets and standard computing environments. In terms of reproducibility, handcrafted radiomics benefit from standardized extraction tools (e.g., PyRadiomics) and feature definitions (e.g., IBSI compliance), whereas deep radiomics pipelines are more sensitive to architectural choices, training parameters, and data variability. Despite these differences, both approaches can be integrated with clinical or genomic data, and fusion models that combine handcrafted and deep features are increasingly used to leverage the strengths of both methodologies.

**Figure 14:**
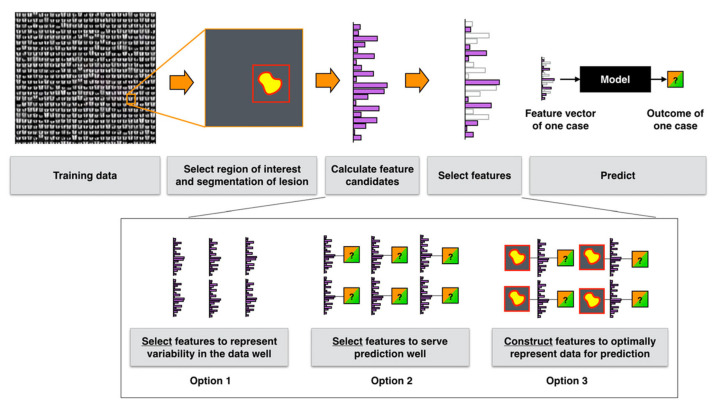
Radiomics workflow [3].

**Figure 15:**
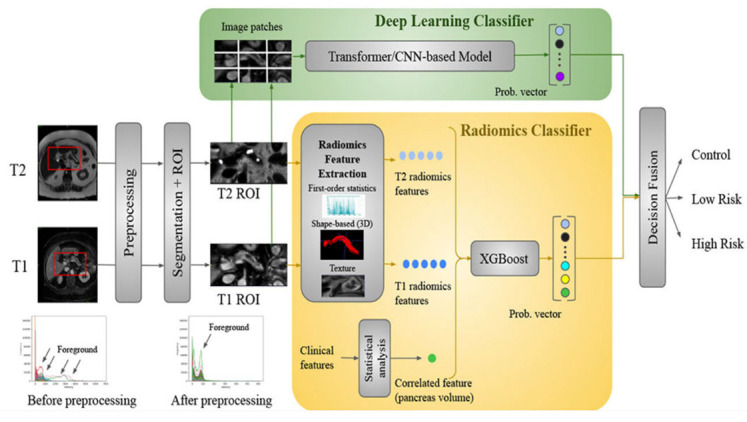
Deep radiomics workflow [140].

Figure 14 illustrates a typical radiomics workflow, whereas Figure 15 shows a deep radiomics approach.

## 6. Discussion

The application of artificial intelligence (AI), deep learning, and radiomics in pancreatic ductal adenocarcinoma (PDAC) detection and management has significantly advanced, yet critical gaps persist across multiple aspects of its clinical translation. While AI-driven approaches have shown promise in disease detection and classification, significant challenges remain in survival prediction, treatment response assessment, and radiogenomics. Addressing these limitations is crucial to bridging the gap between research-based advancements and real-world clinical utility.

One of the most pressing gaps is the limited use of AI models for early PDAC detection. Current studies predominantly focus on diagnosing fully developed tumors rather than identifying subtle pre-diagnostic changes that may indicate early malignancy. Most AI models are trained on datasets consisting of patients with advanced disease, making it difficult to detect small, pre-cancerous lesions that could enable earlier intervention. Furthermore, distinguishing early-stage PDAC from normal or inflamed pancreatic tissue remains a challenge, as radiomics features often overlap between benign and malignant conditions. Future research should focus on refining AI models to recognize minute morphological and textural changes that indicate early-stage PDAC, potentially integrating pre-diagnostic imaging data and circulating biomarkers to enhance accuracy.

Beyond detection and classification, AI-based survival prediction models face significant limitations in generalizability and robustness. Many existing models rely solely on imaging-derived features, ignoring the potential of integrating molecular, clinical, and treatment-related data. The majority of AI-based survival prediction models do not account for the dynamic nature of disease progression, as they typically analyze imaging data from a single time point rather than incorporating longitudinal changes over time. Additionally, overfitting is a common issue, as many models extract thousands of radiomic features, only a fraction of which hold clinical significance. To improve survival prediction, future research should focus on developing multi-modal AI models that integrate radiomics with genomic, proteomic, and metabolomic data. Large-scale, multi-institutional datasets are also needed to enhance the generalizability of AI-driven survival prediction tools.

Another crucial gap in AI-based PDAC research is the limited ability to predict treatment response effectively. Current models struggle to assess how an individual patient will respond to chemotherapy, radiation therapy, or immunotherapy, leading to suboptimal treatment selection. Many AI-driven treatment response models rely solely on pre-treatment imaging, neglecting the importance of tumor biology, immune system interactions, and metabolic changes that influence therapy efficacy. Moreover, most studies define treatment response using broad, retrospective criteria, making it difficult to standardize and validate predictive models. There is a need for AI-driven frameworks that incorporate serial imaging, liquid biopsy markers, and transcriptomic data to predict real-time tumor response to therapy. Additionally, few AI models focus on predicting treatment-related toxicity, which is crucial for personalizing therapy and minimizing adverse effects. Future research should explore AI-driven toxicity prediction models that integrate imaging features with clinical and biochemical markers to optimize treatment decisions.

Radiogenomics, which combines radiomics-based imaging biomarkers with genomic and molecular data, remains an underdeveloped area in PDAC research. Although radiogenomics has shown potential in other cancers, its application in PDAC has been limited due to the lack of large, well-annotated datasets linking imaging phenotypes with genetic alterations. Existing radiogenomics studies focus on a small subset of mutations, such as KRAS, TP53, and CDKN2A, while neglecting other emerging biomarkers, including epigenetic modifications and immune-related gene expression. Furthermore, radiogenomic studies are often conducted in a static manner, failing to account for tumor evolution over time. Future research should emphasize the development of longitudinal radiogenomic models that integrate serial imaging and multi-omics data to track tumor progression and therapy resistance. Additionally, integrating liquid biopsy biomarkers, such as circulating tumor DNA (ctDNA) and exosomal RNA, with imaging features could enhance the ability to detect minimal residual disease and predict recurrence more accurately.

Another key limitation in AI-based PDAC detection is the difficulty in distinguishing PDAC from benign pancreatic lesions, such as chronic pancreatitis and pancreatic cystic neoplasms. Many AI models exhibit a high sensitivity but relatively low specificity, leading to false positives that result in unnecessary biopsies and surgeries. The challenge lies in the similarity of radiomic features between PDAC and benign lesions, which complicates accurate classification. Future research should focus on developing hybrid AI models that integrate imaging with histopathological and molecular profiling to enhance specificity. Additionally, AI models should be designed to function as assistive tools rather than standalone diagnostic systems, incorporating radiologist feedback to refine classification accuracy.

Compounding these technical and scientific gaps is a notable imbalance in data accessibility. Although a handful of public resources, such as The Cancer Imaging Archive (TCIA) or large epidemiological datasets, are available, the majority of studies rely on local, private datasets locked within specific institutions. This widespread preference for private data poses multiple problems: it restricts opportunities for external validation, prevents the uniform benchmarking of AI models, and can perpetuate biases if the private cohorts are demographically narrow or come from a single geographical region. Consequently, many research teams end up training algorithms on data that may not fully represent different imaging protocols, patient populations, or disease subtypes, undermining generalizability. Collaboration across institutions and the willingness to share anonymized imaging and clinical data at scale—admittedly challenging due to privacy regulations—would markedly enhance reproducibility, encourage the standardized evaluation of AI models, and foster a global consensus on best practices for PDAC imaging analysis.

Despite the progress in AI-driven PDAC research, clinical validation remains a major obstacle. Most AI models are trained and tested retrospectively, with only a few studies evaluating their prospective impact in real-world settings. Without clinical trials assessing AI-assisted PDAC diagnosis and prognosis, regulatory approval and widespread adoption remain limited. Healthcare providers require real-world evidence demonstrating that AI can enhance diagnostic accuracy, reduce false positives, and improve clinical workflow efficiency before integrating these tools into standard practice. To bridge this gap, prospective validation studies should be conducted in clinical settings where AI models assist radiologists in real-time decision-making. These studies should assess not only diagnostic accuracy but also the impact of AI on patient outcomes, including early diagnosis rates, survival benefits, and treatment efficacy.

Beyond technical challenges, workflow integration remains a significant barrier to the clinical adoption of AI in pancreatic imaging. Many AI algorithms are developed in research environments with minimal consideration for how they will be implemented in real-world radiology workflows. Current AI tools often require additional steps for image preprocessing, segmentation, and feature extraction, which disrupt the efficiency of clinical workflows. To overcome this challenge, AI models should be designed for seamless integration into radiology information systems and picture archiving and communication systems (PACS). Cloud-based AI platforms that provide automated, real-time image analysis with minimal manual intervention could help facilitate the transition from research to clinical practice.

Finally, ethical concerns and bias in AI-driven PDAC research require further attention. Many AI models are trained on datasets that may not be representative of the global population, leading to potential biases in diagnostic performances across different demographic groups. AI models must be rigorously tested for fairness and robustness to ensure that they perform equitably across diverse patient populations. Additionally, patient data privacy and informed consent must be prioritized in AI research to build trust among clinicians and patients. Future studies should implement fairness-aware AI training techniques and conduct demographic-specific performance analyses to identify and mitigate potential biases in AI-driven pancreatic cancer detection.

Beyond technical and data-related challenges, several real-world factors hinder the clinical adoption of AI tools for PDAC. First, the majority of AI models lack regulatory approval (e.g., FDA, CE marking), which is essential before clinical deployment. Additionally, hospitals face challenges in integrating AI outputs into existing radiology workflows, including PACS/RIS systems, reporting tools, and clinical decision pathways. Radiologists may be hesitant to adopt AI tools that lack transparent reasoning or interpretability, particularly when decisions carry diagnostic or legal consequences. There is also no established reimbursement model for AI-assisted analysis, which limits institutional investment. Finally, liability in the event of misdiagnosis remains legally unclear when AI is involved in decision-making. Addressing these system-level and human-integration barriers is just as crucial as improving technical model performance and will determine whether AI solutions meaningfully impact clinical care in PDAC [10,13,15,142,143].

Table 9 summarizes the key limitations and gaps in the literature alongside possible future directions per defined research category.

It should be highlighted that the transition from traditional radiomics to deep radiomics represents a pivotal advancement in medical image analysis, particularly within PDAC research. Traditional radiomics employs predefined, mathematically derived features that enhance interpretability and reproducibility, while deep radiomics leverages CNNs to learn abstract, data-driven representations directly from images. Though deep radiomics offers a greater modeling flexibility and captures complex patterns, it faces challenges in interpretability and computational demand. Yet, both pipelines report complementary strengths and are increasingly used in fusion models that integrate handcrafted and learned features.

In conclusion, while AI and radiomics have demonstrated an immense potential in PDAC detection, classification, survival prediction, treatment response assessment, and radiogenomics, significant gaps remain in clinical validation, generalizability, interpretability, and workflow integration. Future research should prioritize multi-institutional collaborations, prospective validation studies, and explainable AI techniques to enhance trust and adoption. Additionally, the integration of multi-omics data, liquid biopsies, and real-time AI-driven decision support systems could revolutionize personalized medicine in PDAC. Only through rigorous validation and seamless clinical integration can AI-based PDAC detection and prognosis models transition from experimental research to transformative tools in oncology.

## 7. Conclusions

The present review underscored the remarkable potential of advanced imaging techniques—ranging from radiomics and machine learning to deep learning and fusion-based methodologies—in tackling the multifaceted challenges of pancreatic cancer. By melding detailed radiomic data with powerful computational algorithms, investigators have begun to illuminate subtle tumor phenotypes that were previously undetectable, thereby improving the detection, classification, survival forecasting, and treatment response evaluations. Such insights hold the promise of more accurately stratifying patient risk, tailoring therapeutic interventions, and ultimately moving the field closer to truly individualized care. In order to translate these advances into clinical impact, future work should prioritize external and prospective validation, adopt clinically meaningful evaluation metrics such as specificity and calibration, and design AI tools that can be integrated seamlessly into radiology workflows. Fusion models combining radiomics with deep learning embeddings and multi-modal inputs have shown particular promise and warrant deeper investigation. Addressing data sharing, regulatory standards, and interpretability will also be essential to enable broader adoption. With continued multidisciplinary collaboration and methodological rigor, radiomics-driven AI solutions have the potential to significantly improve outcomes for individuals facing pancreatic cancer.

## Figures and Tables

**Table 1:** Details of studies regarding the use of radiomics and AI methods for disease classification.

Ref.	Dataset	Software/Tool/Prog. Lang. *	Features	ML/DL Model	Results
He et al. (2019) [17]	147 patients (80 PDAC, 67 NF-pNET)	ITK-SNAP, MATLAB, R	7	SVM, Random Forest	Integrated model AUC = 0.884
Xie et al. (2020) [18]	57 patients (MCN vs. MaSCA)	MRIcron	1942	Logistic Regression	AUC = 0.994, Acc. = 98.2%
Mashayekhi et al. (2020) [19]	56 patients (FAP, RAP, and CP)	N/A **	54	IsoSVM	Acc. = 82.1%, AUC = 0.77–0.95
Attiyeh et al. (2018) [20]	103 patients (BD-IPMNs)	N/A	Quantitative imaging features	Random Forest	AUC = 0.79
Liu et al. (2022) [21]	102 patients (PC vs. MFCP)	Pyradiomics	6	LASSO Regression	AUC = 0.973 (train), 0.960 (validation)
Kulali et al. (2018) [22]	30 patients (NF-pNETs and hepatic metastases)	N/A	N/A	N/A	Lower ADC values correlated with high Ki-67 index, MRI predictive tool
Park et al. (2020) [23]	182 patients (89 AIP, 93 PDAC)	N/A	431	Random Forest	AUC = 0.975, Acc. = 95.2%
Wei et al. (2019) [24]	260 patients (SCNs vs. PCNs)	N/A	409	SVM	AUC = 0.837 (validation)
Reinert et al. (2020) [25]	95 patients (53 PDAC, 42 PNENs)	Pyradiomics	92	Logistic Regression	AUC = 0.79, Acc. = 75.8%
Polk et al. (2020) [26]	51 patients (IPMNs)	Healthmyne	39	Logistic Regression	AUC = 0.93 (with ICG criteria)
Flammia et al. (2023) [27]	50 patients (BD-IPMNs)	3D Slicer	107	LASSO Regression	AUC = 0.80–0.99
Benedetti et al. (2021) [28]	39 patients (pancreatic neuroendocrine tumors)	CGITA (MATLAB)	69	N/A	Sphericity AUC = 0.79, tumor volume AUC = 0.79, and voxel-alignment AUC = 0.80–0.85
Tikhonova et al. (2022) [29]	91 patients (PDAC grading)	LifEx	5	LASSO Regression	AUC = 0.75 (grade ≥ 2), AUC = 0.66 (grade 3)
Zhang et al. (2022) [30]	138 patients (MFCP vs. PDAC)	Pyradiomics	LASSO selected features	Logistic Regression	AUC = 0.91 (train), 0.93 (validation)
Kim et al. (2015) [31]	167 lesions (161 patients, pancreatic neuroendocrine neoplasms)	SPSS 18	N/A	N/A	Portal enhancement ratio < 1.1 achieved 92.3% sensitivity, 80.5% specificity
Li et al. (2023) [32]	512 patients (PASC vs. PDAC)	Pyradiomics	N/A	LDA	AUC = 0.94 (validation), sensitivity = 67.57%, and specificity = 97.44%
Chu et al. (2022) [33]	214 patients (PCNs)	N/A	488	Random Forest	AUC = 0.940
Yang et al. (2022) [34]	110 patients (SCNs vs. MCNs)	N/A	N/A	MMRF-ResNet	AUC = 0.98, Acc. = 92.69%
Chen et al. (2021) [35]	89 patients (SCNs vs. PCNs)	N/A	710	Logistic Regression	AUC = 0.960 (train), 0.817 (validation)
Bian et al. (2021) [36]	157 patients (NF-pNETs grading)	N/A	7	LASSO Regression	AUC = 0.775
Ren et al. (2020) [37]	109 patients (MFP vs. PDAC)	N/A	396	Random Forest	Acc. = 93.3%, sensitivity = 92.2%, and specificity = 94.2%
Van der Pol et al. (2019) [38]	71 patients (PNETs vs. RCC metastases)	N/A	Entropy and tumor size	Logistic Regression	AUC = 0.77, sensitivity = 71.4%, and specificity = 79.1%
Zhang et al. (2023) [39]	143 patients (PCNs subtypes)	N/A	1218	Random Forest	Acc. = 80.4% (train), 70.7% (test), and binary models AUC = 0.914–0.926
Chang et al. (2020) [40]	301 patients (PDAC grading)	IBEX	LASSO selected features	SVM	AUC = 0.961 (train), 0.910 (test), and 0.770 (external validation)
Zhu et al. (2013) [41]	388 patients (PC vs. CP)	N/A	105	SVM	Acc. = 94.26%, sensitivity = 96.25%, and specificity = 93.38%
Săftoiu et al. (2012) [42]	258 patients (pancreatic cancer vs. CP)	N/A	Hue histogram features	MLP Neural Network	AUC = 0.94, Acc. = 91.14% (train), and 84.27% (test)
Kang et al. (2015) [43]	44 patients (pRCC vs. pNETs)	N/A	Relative Percentage Washout (RPW)	Threshold-based classification	Acc. = 83.8%, sensitivity = 83.8%, and specificity = 83.9%
Hanania et al. (2016) [44]	53 patients (34 HG-IPMNs, 19 LG-IPMNs)	N/A	360	Logistic Regression	AUC = 0.96, sensitivity = 97%, and specificity = 88%
Proietto Salanitri et al. (2022) [45]	139 patients (normal, LGD, HGD, and adenocarcinoma)	N4 Bias Correction, Gaussian Smoothing, and TensorFlow	N/A	Vision Transformer (ViT)	Acc. = 70%, precision = 67%, and recall = 64%
Gai et al. (2022) [46]	77 patients (33 malignant, 44 benign)	MaZda	1267, reduced to 12	SVM	AUC = 0.750, sensitivity = 60.6%, specificity = 81.8%, and Acc. = 72.7%
Pawlik et al. (2008) [47]	203 patients (multidisciplinary pancreatic cancer review)	N/A	N/A	N/A	Treatment plan changed in 23.6% of cases
Chakraborty et al. (2018) [48]	103 patients (BD-IPMNs)	Scout Liver (Analogic Corp.)	Radiographically inspired (RiFs) + texture features	Random Forest	AUC = 0.81 (with clinical variables), AUC = 0.77 (radiomics alone)
Zhang et al. (2022) [49]	238 patients (156 PDAC, 82 pNET)	LIFEx	48	Gradient Boosting Decision Tree (GBDT) + Random Forest	AUC = 0.971 (train), 0.930 (validation), sensitivity = 0.804, and specificity = 0.973
Ma et al. (2022) [50]	175 patients (151 PC, 24 CP)	MITK, PyRadiomics	1037	LASSO + Logistic Regression	AUC = 0.980, sensitivity = 94.7%, and specificity = 91.7%
Vaiyapuri et al. (2022) [51]	500 CT images (250 tumor, 250 non-tumor)	TensorFlow	N/A	MobileNet + Autoencoder + Emperor Penguin Optimizer (EPO)	Acc. = 99.35%, sensitivity = 99.35%, and specificity = 98.84%
Wang et al. (2022) [52]	139 patients (PNETs grading)	N/A	1133	SVM	AUC = 0.919 (train), 0.875 (validation)
Shi et al. (2020) [53]	66 patients (31 PNETs, 35 SPTs)	ITK-SNAP	195	Logistic Regression	AUC = 0.97 (train), 0.86 (validation), sensitivity = 95%, and specificity = 91.67%
Ren et al. (2019) [54]	109 patients (30 MFP, 79 PDAC)	AnalysisKit (GE Healthcare)	396	Logistic Regression	AUC = 0.98, sensitivity = 94%, and specificity = 92%
Yang et al. (2019) [55]	78 patients (53 SCAs, 25 MCAs)	LIFEx	22 (2 mm slices)/18 (5 mm slices)	Random Forest	AUC = 0.77 (train, 2 mm), 0.66 (validation, 2 mm), and 0.75 (validation, 5 mm)
Li et al. (2019) [56]	206 patients (64 IPMNs, 35 MCNs, 66 SCNs, and 41 SPTs)	TensorFlow	N/A	DenseNet CNN	Acc. = 72.8% (outperforms manual reading at 48.1%)
Bevilacqua et al. (2021) [57]	51 patients (PanNETs, G1 vs. G2)	ImageJ 1.53f	N/A	Logistic Regression	AUC = 0.90 (best model), sensitivity = 88%, and specificity = 89%
Gu et al. (2019) [58]	138 patients (PNETs, G1 vs. G2/3)	N/A	853	Random Forest	AUC = 0.974 (train), 0.902 (validation)
Kuwahara et al. (2019) [59]	206 patients (50 for deep learning analysis, 3970 EUS images)	TensorFlow	N/A	ResNet50 CNN	AUC = 0.98, sensitivity = 95.7%, specificity = 92.6%, and Acc. = 94.0%
Tobaly et al. (2020) [60]	408 patients (181 LGD, 128 HGD, and 99 invasive carcinoma)	MedSeg, PyRadiomics	107	LASSO + Logistic Regression	AUC = 0.84 (train), 0.71 (validation)
Li et al. (2018) [61]	127 patients (50 PDAC, 77 pNET)	FireVoxel	Histogram-based texture features	Threshold-Based Classification	AUC = 0.887, sensitivity = 90%, and specificity = 80%
Hernandez-Barco et al. (2023) [62]	575 patients (IPMN surgical cases)	N/A	18 clinical and imaging variables	Linear SVM	AUC = 0.82, Acc. = 77.4%, sensitivity = 83%, and specificity = 72%
Cui et al. (2021) [63]	202 patients (BD-IPMN grading)	ITK-SNAP, MITK	1312	LASSO + Logistic Regression	AUC = 0.903 (train), 0.884 (validation 1), and 0.876 (validation 2)
Guo et al. (2018) [64]	42 patients (28 PDAC, 14 PNEC)	MATLAB	Contrast ratio + texture features	Threshold-Based Classification	AUC = 0.98–0.99 (contrast ratio), 0.71–0.72 (texture features)
Tong et al. (2022) [65]	558 patients (PDAC vs. CP)	ResNet-50 (DL model)	N/A	Deep Learning (CNN)	AUC = 0.986 (train), 0.978 (internal validation), and 0.953 (external validation)
Liang et al. (2022) [66]	193 patients (99 SCA, 55 MCA, and 39 IPMN)	ITK-SNAP	1067	SVM, CNN (Hybrid Model)	AUC = 0.916 (SCA), 0.973 (MCA vs. IPMN)

* Note: “Software/Tool/Prog. Lang.” refers to the primary platform, package, or programming environment used for radiomic feature extraction or model implementation (e.g., PyRadiomics, Python, MATLAB, and LIFEx). ** Not available.

**Table 2:** Details of studies regarding the use of radiomics and AI methods for disease detection.

Ref.	Dataset	Software/Tool/Prog. Lang. *	Features	ML/DL Model	Results
Korfiatis et al. (2023) [67]	696 PC, 1080 control CTs	TensorFlow 2.3.1	N/A **	Modified ResNet + Attention Modules	AUROC = 0.97, Acc. = 92%
Alizadeh Savareh et al. (2020) [68]	671 miRNA profiles	MATLAB 2019	N/A	ANN + PSO + NCA	Acc. = 93%, Sensitivity = 93%, and Specificity = 92%
D’Onofrio et al. (2021) [69]	91 MRI scans	MeVisLab, MATLAB	ADC Histogram (Entropy-based)	N/A	Acc. = 89.01%, Sensitivity = 90.77%, and Specificity = 84.62%
Xia et al. (2023) [70]	662 PDAC, 450 PanNETs, 458 cysts, and 846 normal	N/A	N/A	3D U-Net	Sensitivity = 97%, Specificity = 99%, and DSC = 87%
Chen et al. (2023) [71]	10,673 patients (8 cancers + 1055 controls)	nnUNet, CTLabler, and ITK-SNAP	N/A	CancerUniT Transformer	Sensitivity = 93.3%, Specificity = 81.7%, and DSC = 62.8%
Zhang et al. (2020) [72]	2890 pancreatic CTs	TensorFlow	N/A	Faster R-CNN + AFPN	AUC = 0.9455, Acc. = 90.18%, Sensitivity = 83.76%, and Specificity = 91.79%
Chen et al. (2021) [73]	436 PDAC, 479 control CTs	PyRadiomics	88 features	XGBoost 2.1.0	Acc. = 95.0%, Sensitivity = 94.7%, and Specificity = 95.4%
Chen et al. (2022) [74]	546 PC, 733 control	TensorFlow	N/A	Ensemble CNNs	AUC = 0.96, Sensitivity = 89.9%, and Specificity = 95.9%
Chu et al. (2019) [75]	190 PDAC, 190 controls	Velocity 3.2.0	478 features	Random Forest	Acc. = 99.2%, AUC = 99.9%, Sensitivity = 100%, and Specificity = 98.5%
Liu et al. (2019) [76]	238 PC, 4385 CTs	N/A	N/A	Faster R-CNN + VGG16	AUC = 0.9632
Abel et al. (2021) [77]	221 CTs, 543 cysts	SPSS Statistics, nnUNet	N/A	CNN (2-step nnU-Net)	Sensitivity = 78.8%, Specificity = 96.2%
Ozkan et al. (2016) [78]	332 EUS images (202 PC, 130 non-PC)	MATLAB	122 features	ANN	Acc. = 87.5%, Sensitivity = 83.3%, and Specificity = 93.3%
Zhang et al. (2020) [79]	573 PDAC, 153 adjacent normal, 10 pancreatitis, and 74 normal	LibSVM v3.23	N/A	SVM	Acc. = 98.77%, Sensitivity = 98.65%, and Specificity = 100%
Deng et al. (2021) [80]	119 MRI scans (PDAC vs. MFCP)	IBEX	N/A	SVM	AUC = 0.997 (Training), 0.962 (Validation)
Javed et al. (2022) [81]	108 CTs	ITK-SNAP	N/A	Naïve Bayes + RFE	Acc. = 89.3%, Sensitivity = 86%, and Specificity = 93%
Qureshi et al. (2022) [82]	108 CTs (36 pre-diagnostic PDAC, 36 PC, and 36 control)	ITK-SNAP	4000 features	Naïve Bayes	Acc. = 86%
Park et al. (2022) [83]	852 training, 603 and 589 test patients	nnU-Net	N/A	3D CNN	AUC = 0.91
Mukherjee et al. (2022) [84]	155 pre-diagnostic CTs, 265 normal	3D Slicer, PyRadiomics	88 features	SVM	Acc. = 92.2%, AUC = 0.98
Chen et al. (2023) [85]	227 non-CP, 70 CP	MATLAB	111 features	SVM	AUC = 0.99
Frøkjær et al. (2020) [86]	77 CP, 22 controls	3D Slicer	851 features	Bayes Classifier	Acc. = 98%, Sensitivity = 97%, and Specificity = 100%
Gonoi et al. (2017) [87]	9 PDAC, 103 controls	N/A	N/A	Kaplan–Meier survival analysis	Identified Early Imaging Markers
Si et al. (2021) [88]	143,945 CT images (319 patients), 107,036 test images (347 patients)	TensorFlow	N/A	ResNet18 (pancreas detection), U-Net32 (segmentation), and ResNet34 (classification)	AUC = 0.871, Acc. = 82.7%, and F1-score = 88.5%
Ma et al. (2020) [89]	7245 CT images (412 patients)	N/A	N/A	CNN	Acc. = 95.47% (Plain Scan), 95.76% (Arterial Phase), Sensitivity = 91.58%, and Specificity = 98.27%
Hsieh et al. (2018) [90]	1,358,634 patients (3092 pancreatic cancer cases)	Python 3.7 (scikit-learn), TensorFlow	22 clinical variables	Logistic Regression, ANN	AUC = 0.727 (LR), 0.605 (ANN), and F1-score = 0.997
Muhammad et al. (2019) [91]	800,114 respondents (NHIS and PLCO datasets), 898 pancreatic cancer cases	N/A	18 personal health features	Artificial Neural Network (ANN)	AUC = 0.86 (Training), 0.85 (Testing), Sensitivity = 87.3%, and Specificity = 80.7%
Boursi et al. (2017) [92]	109,385 new-onset diabetes patients (390 diagnosed with PDAC)	N/A	N/A	Logistic Regression	AUC = 0.82, Specificity = 94%, and Sensitivity = 44.7%
Appelbaum et al. (2021) [93]	594 PDAC cases, 100,787 controls (training), 408 PDAC cases, and 160,185 controls (validation)	L2-regularized logistic regression, neural network	ICD codes, comorbidities, and medication history	Logistic Regression, Neural Network	AUC = 0.71 (training), 0.68 (validation)
Das et al. (2008) [94]	110 normal pancreas, 99 CP, and 110 PC (EUS images)	ImageJ	228 features reduced to 11	Artificial Neural Network (ANN)	AUC = 0.93, Sensitivity = 93%, and Specificity = 92%
Urman et al. (2020) [95]	129 bile samples (57 PDAC, 36 CCA, and 36 benign)	UHPLC-MS, HPLC-MS/MS	N/A	Neural Network	AUC = 1.00
Liu et al. (2020) [96]	370 PC, 320 controls	N/A	N/A	CNN	AUC = 1.00 (Local), AUC = 0.83 (External)
Săftoiu et al. (2008) [97]	68 patients (32 PC, 11 CP, 22 normal, and 3 PNET)	ImageJ	228 features reduced to 11	Multilayer Perceptron (MLP) Neural Network	AUC = 0.932, Sensitivity = 91.4%, Specificity = 87.9%, and Acc. = 89.7%

* Note: “Software/Tool/Prog. Lang.” refers to the primary platform, package, or programming environment used for radiomic feature extraction or model implementation (e.g., PyRadiomics, Python, MATLAB, and LIFEx). ** Not available.

**Table 3:** Details of studies regarding the use of radiomics and AI methods for survival prediction.

Ref.	Dataset	Software/Tool/Prog. Lang. *	Features	ML/DL Model	Results
Cheng et al. (2019) [98]	41 patients (unresectable PDAC, contrast-enhanced CT)	TexRAD	Mean intensity, entropy, skewness, kurtosis, and SD	None	Higher SD associated with longer OS (*p* = 0.04)
Khalvati et al. (2019) [99]	98 patients (resectable PDAC, contrast-enhanced CT)	PyRadiomics v2.0.1	410 extracted, 277 robust	Cox proportional-hazards regression	HR = 1.56, *p* = 0.005
Yun et al. (2018) [100]	88 patients (pancreatic head cancer, contrast-enhanced CT)	In-house software	Histogram and GLCM texture features	None	Lower SD and contrast are associated with poor DFS
Eilaghi et al. (2017) [101]	30 patients (resectable PDAC, contrast-enhanced CT)	MATLAB (R2015a)	5 GLCM texture features	None	Dissimilarity (*p* = 0.045) and IDN (*p* = 0.046) significant for OS
Miyata et al. (2020) [102]	183 patients (resected PDAC, tumor markers)	JMP v12 (SAS Institute)	None (clinical markers only)	None	High Pre-TI associated with worse OS (HR = 2.27, *p* < 0.0001)
Healy et al. (2022) [103]	352 training, 215 validation (resectable PDAC, contrast-enhanced CT)	PyRadiomics v3.0	IBSI-compliant radiomics features	LASSO Cox regression	C-index = 0.545 (radiomics), 0.497 (clinical)
Attiyeh et al. (2018) [104]	161 patients (resectable PDAC, contrast-enhanced CT)	MATLAB (R2015a)	CT texture features	Cox proportional-hazards regression	C-index = 0.69 (radiomics), 0.74 (clinical)
Xie et al. (2020) [105]	220 patients (resectable PDAC, contrast-enhanced CT)	R software	300 radiomics features	LASSO regression	AUC = 0.87 (training), 0.85 (validation)
Kim et al. (2019) [106]	45 patients (PDAC post-neoadjuvant therapy, contrast-enhanced CT)	MISSTA	GLCM texture features	None	Higher entropy (HR = 0.159, *p* = 0.005) predicted longer OS
Choi et al. (2019) [107]	66 patients (PDAC, MRI T2-weighted imaging)	TexRAD	Histogram and GLCM features	None	Higher entropy (*p* = 0.002) correlated with worse OS
Parr et al. (2020) [108]	74 patients (PDAC, SBRT, and contrast-enhanced CT)	3D Slicer	800+ radiomics features	None	Radiomics model outperformed clinical (C-index = 0.66)
Cozzi et al. (2019) [109]	100 patients (PDAC, SBRT, and contrast-enhanced CT)	LifeX	Radiomics features	Cox regression	C-index = 0.73–0.75 for OS prediction
Tang et al. (2019) [110]	303 patients (resectable PDAC, and MRI multiparametric)	ITK-SNAP, A.K.	328 radiomics features	LASSO logistic regression	AUC = 0.87 (training), 0.85 (validation)
Wang et al. (2022) [111]	184 patients (resectable PDAC, contrast-enhanced CT)	PyRadiomics	1409 extracted, LASSO selected	Cox regression	C-index = 0.74 (radiomics), 0.68 (clinical)
Chakraborty et al. (2017) [112]	35 patients (PDAC, contrast-enhanced CT)	MATLAB (R2015a)	255 texture features	Naïve Bayes classifier	AUC = 0.90 (leave-one-out), 0.80 (3-fold CV), and Acc. = 82.86%
Kaissis et al. (2019) [113]	102 training, 30 validation (PDAC, diffusion-weighted MRI)	PyRadiomics	ADC-based radiomic features	Random Forest	AUC = 0.90 (survival prediction), 89% acc. for tumor subtype classification
Zhang et al. (2020) [114]	68 training, 30 validation (resectable PDAC, contrast-enhanced CT)	None	None	CNN (6-layer)	C-index = 0.651, IPA = 11.81%
Shi et al. (2021) [115]	299 patients (resectable PDAC, contrast-enhanced CT)	A.K. (GE Healthcare), ITK-SNAP	1409 extracted, LASSO selected	Cox regression	C-index = 0.74 (radiomics), 0.68 (clinical)
Rezaee et al. (2016) [116]	616 patients (IPMN, pancreatic resection)	None	None	None	High-grade dysplasia linked to increased PDAC risk, median OS = 92 months

* Note: “Software/Tool/Prog. Lang.” refers to the primary platform, package, or programming environment used for radiomic feature extraction or model implementation (e.g., PyRadiomics, Python, MATLAB, and LIFEx).

**Table 4:** Details of studies regarding the use of radiomics and AI methods for treatment response.

Ref.	Dataset	Software/Tool/Prog. Lang. *	Features	ML/DL Model	Results
Abraham et al. (2021) [117]	517 patients (105 training, 412 validation, and 55 FOLFIRI control)	N/A **	67 gene signatures	Bayesian Regularization Neural Network	OS HR = 0.629 (*p* = 0.04) for FOLFOX, 0.483 (*p* = 0.02) for FOLFOXIRI
Ciaravino et al. (2018) [118]	31 patients (17 downstaged, 14 progression)	MaZda	Histogram, texture, and kurtosis	N/A	Kurtosis change (*p* = 0.0046) is significant in responders
Mu et al. (2020) [119]	583 patients (513 training, 70 validation)	3D Slicer, Keras, Python	N/A	CNN	AUC = 0.85 (train), 0.81 (val), and 0.89 (test)
Nasief et al. (2019) [120]	90 patients, 2520 daily CT scans	IBEX	1300+ features	Bayesian Regularization Neural Network	AUC = 0.94
Nasief et al. (2020) [121]	24 patients (672 CT datasets)	IBEX	1300+ features	Regression Model	C-index = 0.87, HR = 0.58
McClaine et al. (2010) [122]	29 patients (26 neoadjuvant, 12 resected)	N/A	N/A	N/A	Median survival: 15.5 months (unresected) vs. 23.3 months (resected), *p* = 0.015
Yue et al. (2017) [123]	26 PA patients (19 external-beam RT, 7 SBRT)	N/A	Texture features from PET	Lasso Regression, Cox Model	OS = 29.3 months (low-risk) vs. 17.7 months (high-risk)
Cassinotto et al. (2013) [124]	80 patients (38 neoadjuvant)	N/A	N/A	N/A	CT acc. lower after neoadjuvant (58% vs. 83%, *p* = 0.039)
Chen et al. (2017) [125]	20 patients, daily CT scans	N/A	Mean CT number, volume, and skewness	N/A	MCTN decrease (−4.7 HU, *p* < 0.001) correlated with response
Rigiroli et al. (2021) [126]	194 PDAC patients (148 neoadjuvant)	Siemens SyngoVia Frontier Radiomics	1695 features	Logistic Regression	AUC = 0.71, sensitivity = 62%, and specificity = 77%
Bian et al. (2020) [127]	181 PDAC patients	N/A	1029 features (portal phase CT)	Logistic Regression	AUC = 0.75, sensitivity = 64.8%, and specificity = 74%
Gregucci et al. (2022) [128]	37 locally advanced PDAC patients	Imaging Biomarker Explorer	27 radiomic features	Logistic Regression	AUC = 0.851

* Note: “Software/Tool/Prog. Lang.” refers to the primary platform, package, or programming environment used for radiomic feature extraction or model implementation (e.g., PyRadiomics, Python, MATLAB, and LIFEx). ** Not available.

**Table 5:** Details of studies regarding the use of radiomics and AI methods for radiogenomics.

Ref.	Dataset	Software/Tool/Prog. Lang. *	Features	ML/DL Model	Results
McGovern et al. (2018) [129]	121 PanNET patients	N/A **	Tumor size, shape, necrosis, vascular invasion, and pancreatic duct dilatation	Multivariate Logistic Regression	AUC = 0.58, *p* = 0.006
Attiyeh et al. (2019) [130]	35 PDAC patients	Scout Liver Software, MATLAB	255 features (GLCM, RLM, LBP, FD, IH, and ACM)	Fuzzy Minimum-Redundancy-Maximum-Relevance (fMRMR)	R^2^ = 0.731, RMSE = 19.5
Lim et al. (2020) [131]	48 PDAC patients	MIM v6.4, MATLAB (CGITA toolbox)	35 PET-based radiomic features	Logistic Regression	AUC = 0.806 (KRAS), 0.727 (SMAD4)
Iwatate et al. (2020) [132]	107 PDAC patients	PyRadiomics v2.2.0	2074 features (early- and late-phase CT)	XGBoost	AUC = 0.795 (p53), 0.683 (PD-L1)
Tang et al. (2024) [133]	205 patients (151 internal, 54 CPTAC-PDAC)	ITK-SNAP, PyRadiomics	1239 features	StepGBM + Elastic Net	AUC = 0.84 (train), 0.85 (val)
Hinzpeter et al. (2022) [134]	47 PDAC patients	LIFEx v6.30	Multiple HU and texture-based features	Logistic Regression	Youden Index: 0.67 (TP53), 0.56 (KRAS), and 0.50 (SMAD4, CDKN2A)
Iwatate et al. (2022) [135]	107 PDAC patients (RNA-seq: 12)	ITK-SNAP	3748 radiomic features	XGBoost	AUC = 0.697 (ITGAV), *p* = 0.048 (OS correlation)

* Note: “Software/Tool/Prog. Lang.” refers to the primary platform, package, or programming environment used for radiomic feature extraction or model implementation (e.g., PyRadiomics, Python, MATLAB, and LIFEx). ** Not available.

**Table 6:** Details of studies regarding the use of radiomics and AI methods for deep radiomics fusion models.

Ref.	Dataset	Software/Tool/Prog. Lang. *	Features	ML/DL Model	Results
Dmitriev et al. (2017) [136]	134 patients (4 pancreatic cyst types)	Scikit-learn, Keras (NVIDIA Titan X GPU)	14 radiomic features + CNN deep features	Random Forest + CNN (Bayesian Fusion)	Acc = 83.6%
Ziegelmayer et al. (2020) [137]	86 patients (44 AIP, 42 PDAC)	PyRadiomics, pretrained VGG19	1411 radiomic features + 256 deep features	Extremely Randomized Trees	AUC = 0.90, Sens = 89%, and Spec = 83%
Zhang et al. (2021) [138]	98 PDAC patients (68 training, 30 validation)	PyRadiomics (v2.0.0) + 8-layer CNN (LungTrans)	1428 radiomic features + 35 deep features	Risk Score-Based Fusion (Random Forest)	AUC = 0.84
Wei et al. (2023) [139]	112 patients (64 PDAC, 48 AIP) with 18F-FDG PET/CT	PyRadiomics + VGG11 CNN	Radiomics (texture, histogram) + CNN deep features	Multidomain Fusion Classifier	AUC = 96.4%, Acc = 90.1%, Sens = 87.5%, and Spec = 93.0%
Yao et al. (2023) [140]	246 multi-center MRI scans (IPMN risk stratification)	nnUNet, multiple CNNs (DenseNet, ViT, etc.)	107 radiomic features + deep CNN/ViT + clinical	Weighted Averaging-Based Fusion	Acc = 81.9%
Vétil et al. (2023) [141]	2319 training + 1094 test CT scans (9 centers)	PyRadiomics + Variational Autoencoder (VAE)	Handcrafted radiomics + MI-minimized deep features	Logistic Regression (Fusion)	+1.13% AUC improvement over radiomics alone

* Note: “Software/Tool/Prog. Lang.” refers to the primary platform, package, or programming environment used for radiomic feature extraction or model implementation (e.g., PyRadiomics, Python, MATLAB, and LIFEx).

**Table 7:** Datasets used in each category of the selected literature.

Clinical Application	Public Dataset
Disease Detection	The Cancer Imaging Archive CPTAC-PDAC CT set
	4 × GEO expression profiles
	NIH Pancreas-CT dataset
	Public dataset—No name
	16 × GEO expression profiles
	Longitudinal Cohort of Diabetes Patients (LHDB)–Taiwan NHI
	National Health Interview Survey (NHIS)
	The Health Improvement Network (THIN) primary-care database
	Medical Segmentation Decathlon (MSD)–pancreas task
	TCIA pancreas-CT collection (NTU study)
Radiogenomics	cBioPortal PDAC sequencing cohorts
	CPTAC-PDAC multi-omics set
Disease Classification	N/A *
Survival Prediction	N/A
Treatment Response	N/A
Deep Radiomics Fusion	N/A

* No public sources; all studies used only private data.

**Table 8:** Number and types of features alongside the used methodology in each clinical application.

Clinical Application	Number of Features	Types of Features	Model Algorithm	Performance (Typical Metrics: AUC/Acc.)	Primary Purpose
Disease Detection	88–1500+ (radiomics) or additional deep features	Handcrafted features such as shape, first-order intensity, GLCM texture, wavelet, and morphological descriptors; CNN embeddings capture abstract patterns.	3D CNNs (ResNet variants, U-Net, and Faster R-CNN), patch-based CNNs, Random Forests, and SVM ensembles	AUCs: 0.90–0.97; Acc.: ~90–95%	Automatic detection of pancreatic cancer and differentiation from normal tissue or benign lesions.
Disease Classification	22–1000+ (often reduced to 10–40 key features)	Texture descriptors (e.g., GLCM, GLRLM, fractal, and LBP), intensity histograms, and morphological indicators (volume, diameter, and shape factors).	SVMs, Random Forests, Logistic Regression, deep CNN classifiers, and ensemble methods (e.g., gradient boosting)	AUCs: 0.80–0.98; Acc.: ~85–95%	Differentiating among pancreatic pathologies such as PDAC, pancreatitis, PanNETs, and various cystic neoplasms.
Survival Prediction	30–1400+ (often reduced to <10 or fused with clinical data)	Texture features (entropy, dissimilarity), first-order statistics, and morphological descriptors; often combined with clinical biomarkers (CA19-9, CEA).	Cox proportional hazards models, Random Forest Survival, Bayesian neural networks, and deep CNN survival models	C-index: ~0.65–0.75 (often >0.70); AUC: ~0.80–0.90 for early response	Predicting overall/disease-free survival and risk stratification in pancreatic cancer patients.
Treatment Response	100–1000 (including delta-radiomics from daily scans)	Delta changes in texture (kurtosis, skewness, and entropy) and morphological features (tumor shrinkage, density changes) over time.	Logistic or Cox regression models, and deep CNN-based segmentation networks	AUC/Acc.: ~0.75–0.85	Evaluating early treatment response and efficacy in neoadjuvant, chemoradiation, or SBRT settings.
Radiogenomics	2000+–3000+	Comprehensive radiomics encompassing texture, shape, and intensity measures correlated with genomic data (e.g., KRAS, TP53, SMAD4 mutations, and gene expression).	Random Forest, XGBoost, and SVM with recursive feature elimination and importance ranking	AUC: ~0.70–0.80	Linking imaging phenotypes to molecular/genetic profiles to guide precision oncology.
Deep Radiomics Fusion	14–2000 (handcrafted) plus ~256 deep features	Combination of interpretable handcrafted radiomics (GLCM, wavelet, etc.) and deep CNN embeddings capturing high-level image patterns.	Ensemble methods (Bayesian fusion, Random Forest, and Logistic Regression) with mutual information minimization techniques	Improvement of ~2–5% (often achieving AUC up to 0.90+)	Integrating complementary imaging biomarkers to boost performance in detection, classification, and survival prediction.

**Table 9:** Limitations, gaps in the literature, and future research directions for different categories in focus.

Category	Key Limitations/Gap	Possible Future Directions
Early Detection	Limited focus on early-stage PDAC; poor differentiation from benign/inflamed tissue	Develop models using pre-diagnostic data and biomarkers; enhance sensitivity to subtle features
Survival Prediction	Lack of multi-modal data; single time point analysis; and overfitting	Use longitudinal data; integrate clinical, genomic, proteomic, and metabolomic information
Treatment Response	Inability to predict individual therapy outcomes; limited data types used	Incorporate serial imaging, transcriptomics, and immune/metabolic markers
Radiogenomics	Narrow mutation scope; no temporal tracking; and limited datasets	Develop longitudinal radiogenomic models; include liquid biopsy and multi-omics data
Specificity and False Positives	Overlap with benign lesions; high false-positive rates	Combine imaging with histopathology/molecular profiling; design models with radiologist feedback
Data Availability	Predominant use of private data; poor reproducibility and benchmarking	Promote multi-institutional data sharing; build large, diverse, and publicly available datasets
Clinical Validation	Mostly retrospective studies; limited real-world testing	Conduct prospective trials; measure impact on diagnostic accuracy and patient outcomes
Workflow Integration	Models not designed for clinical systems; workflow disruptions	Develop plug-and-play AI tools integrated with PACS/RIS; utilize cloud-based real-time platforms
Bias and Ethics	Lack of fairness testing; biased datasets; and privacy issues	Ensure demographic diversity; apply fairness-aware training; and enforce strong data governance
Regulatory and Adoption Barriers	Lack of regulatory approvals (e.g., FDA, CE); unclear reimbursement; and clinician trust issues	Establish clear validation pathways and regulatory standards; include explainability mechanisms; align with reimbursement models; and promote clinician–AI co-pilot systems
